# Friedel–Crafts approach to the one-pot synthesis of methoxy-substituted thioxanthylium salts

**DOI:** 10.3762/bjoc.15.208

**Published:** 2019-09-05

**Authors:** Kenta Tanaka, Yuta Tanaka, Mami Kishimoto, Yujiro Hoshino, Kiyoshi Honda

**Affiliations:** 1 Graduate School of Environment and Information Sciences, Yokohama National University, Tokiwadai, Hodogaya-ku, Yokohama 240-8501, Japan

**Keywords:** Friedel–Crafts reaction, metal-free conditions, one-pot synthesis, photoredox catalyst, thioxanthylium salt

## Abstract

An efficient synthesis of methoxy-substituted thioxanthylium salts has been developed. The reaction of diaryl sulfides with benzoyl chlorides in the presence of TfOH smoothly proceeded to give the desired thioxanthylium salts in good yields. In their UV–vis spectra, the maximum absorption wavelengths of methoxy-functionalized thioxanthylium salts were observed at around 460 nm, which show a drastic red shift compared to the parent thioxanthylium salts. The present reaction provides a versatile access to functionalized thioxanthylium salts, and therefore it constitutes a promising tool for the synthesis of biologically and photochemically active molecules.

## Introduction

**Scheme 1:**
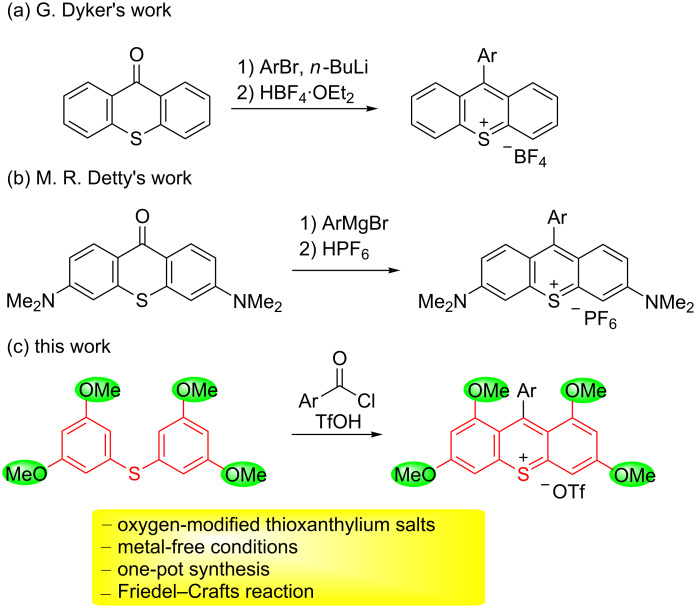
The representative synthesis of thioxanthylium salts.

Thioxanthylium salts are one of the important structural motifs found in biologically active compounds and photochemical materials [1–8]. Owing to these useful properties, several research groups have developed methodologies to synthesize them. The typical synthetic methods for thioxanthylium salts include the reaction of thioxanthone with aryl bromide in the presence of *n*-butyllithium or Grignard reagents followed by dehydration by acids such as hexafluorophosphoric acid (Scheme 1a and 1b) [3–4,9–10], oxidation of thioxanthene in the presence of PbO_2_ followed by dehydration by tetrafluoroboric acid [1], the reaction of 4,4’-bis(dimethylamino)diphenylmethane with sulfur in the presence of ZnCl_2_ [11], and the ring-closure reaction of diaryl sulfide in the presence of a Lewis acid such as SnCl_4_ and AlCl_3_ [12–14]. While these reactions were proven to be useful, they require the use of stoichiometric amounts of metals and/or toxic metal reagents. Moreover, there are only a few methods for the synthesis of thioxanthylium salts despite their useful active properties. Thus, developing efficient synthetic routes and more economic approaches is highly desirable. In addition, only amino groups were introduced to the thioxanthylium core except at the 9-position of the thioxanthylium salt (Scheme 1b), and the physical properties of these substituted compounds have not been examined.

We have developed the synthesis of multisubstituted condensed heterocyclic compounds in the presence of an acid catalyst [15–23]. More recently, we have reported the design and synthesis of thioxanthylium organophotoredox catalysts, which can work under green light irradiation [24–25]. In the course of this study, we found that these thioxanthylium photocatalysts efficiently oxidized styrene derivatives such as *trans*-anethole, and promoted radical cation Diels–Alder reactions. Based on the background mentioned above, in order to expand the utility of the synthesis of thioxanthylium salts and investigate their physical properties, we report the Friedel–Crafts approach as an efficient synthetic method of methoxy-substituted thioxanthylium salts (Scheme 1c).

## Results and Discussion

Initially, we screened the reaction of bis(3,5-dimethoxyphenyl) sulfide (**1a**) with benzoyl chloride (**2a**) in the presence of Brønsted acids in chlorobenzene at several temperatures (Table 1). When we used a strong Brønsted acid such as trifluoromethanesulfonic acid (TfOH) at room temperature, the desired thioxanthylium salt **3a** was obtained with 21% yield while other typical Brønsted acids did not work efficiently (Table 1, entries 1–8) [2,9–10]. At 60 °C, the yield effectively improved to 60% (Table, entry 9). Moreover, when the reaction temperature was increased to 90 °C, 120 °C, and reflux, higher yields were observed, especially under reflux conditions providing the product **3a** in 82% yield (Table 1, entries 10–12). Decreasing the amount of TfOH did not improve the yield (Table 1, entry 13). It is suggested that the cyclization and dehydration were efficiently promoted at high temperature. Fortunately, when the reaction was carried out with benzoic acid, which is a more easily available substrate in comparison with benzyl chloride, the desired product was obtained in good yield. It was found that the reaction can be applied to not only benzoyl chloride but also to benzoic acid.

**Table 1:** Optimization of the reaction conditions^a^.

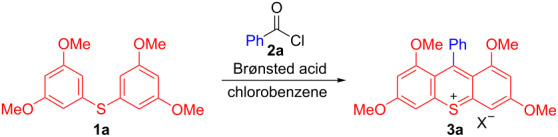			

Entry	Brønsted acid^b^	Temperature (°C)	Yield (%)

1	H_3_PO_4_	rt	0
2	HCl	rt	0
3	TsOH	rt	0
4	MsOH	rt	0
5	HBF_4_	rt	0
6	HPF_6_	rt	0
7	HClO_4_	rt	traces
8	TfOH	rt	21
9	TfOH	60	60
10	TfOH	90	72
11	TfOH	120	75
12	TfOH	reflux	82
13^c^	TfOH	reflux	78
14^d^	TfOH	reflux	72

^a^All reactions were carried out with **1a** (0.25 mmol), **2a** (0.75 mmol), acid (3.0 equiv) in chlorobenzene (5.0 mL) for 1 h under N_2_. ^b^H_3_PO_4_ (85% aq), HCl (0.5 M in MeOH), HBF_4_ (42% aq), HPF_6_ (65% aq), HClO_4_ (70% aq). ^c^TfOH (2.0 equiv) was used. ^d^benzoic acid (0.75 mmol) was used instead of benzoyl chloride (**2a)**.

**Figure 1:**
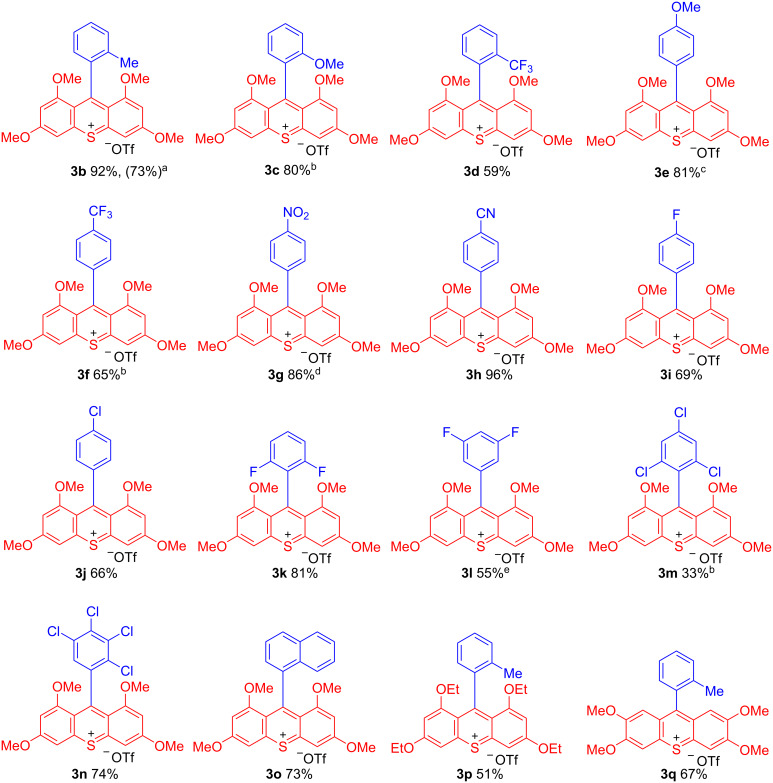
The generality of diaryl sulfide **1** and benzoyl chloride **2**. ^a^The reaction was carried out with **1a** (2.0 mmol), **2a** (6.0 mmol), TfOH (3.0 equiv) in chlorobenzene (40.0 mL) at reflux for 1 h under N_2_. ^b^**2** (2.0 equiv) and TfOH (2.0 equiv) were used at 120 °C. ^c^**2** (2.0 equiv) and TfOH (2.0 equiv) were used. ^d^20 h. ^e^2 h.

With the optimized conditions in hand, we investigated the generality of diaryl sulfide **1** and benzoyl chloride **2** (Figure 1). *o*-Toluoyl chloride smoothly afforded the desired product **3b** in excellent yield. Moreover, the reaction was performed on the 2 mmol scale to furnish the desired product in 73% yield, suggesting that the reaction can be applied to large scale conditions. In addition, 2-methoxy and 2-trifluoromethyl-functionalized benzoyl chloride can be applied to the reaction (**3c**,**d**). The 4-methoxy group was also tolerated in the reaction (**3e**). Substrates with strong electron-withdrawing groups such as 4-trifluoromethyl, 4-nitro and 4-cyano groups reacted with moderate to excellent yields (**3f**–**h**). The benzoyl chlorides bearing a variety of halogens were suitable for this reaction (**3i**–**n**). Although naphthalene is a sterically large group, the reaction proceeded smoothly (**3o**). The diaryl sulfide with ethoxy substituents furnished the corresponding product in moderate yield (**3p**). Interestingly, when bis(3,4-dimethoxyphenyl) sulfide was used as a substrate, the reaction proceeded to afford the desired 2,3,6,7-tetramethoxy-substituted thioxanthylium salt (**3q**). It was found that the present reaction can be applied to various benzoyl chlorides bearing either electron-donating or electron-withdrawing groups.

**Figure 2:**
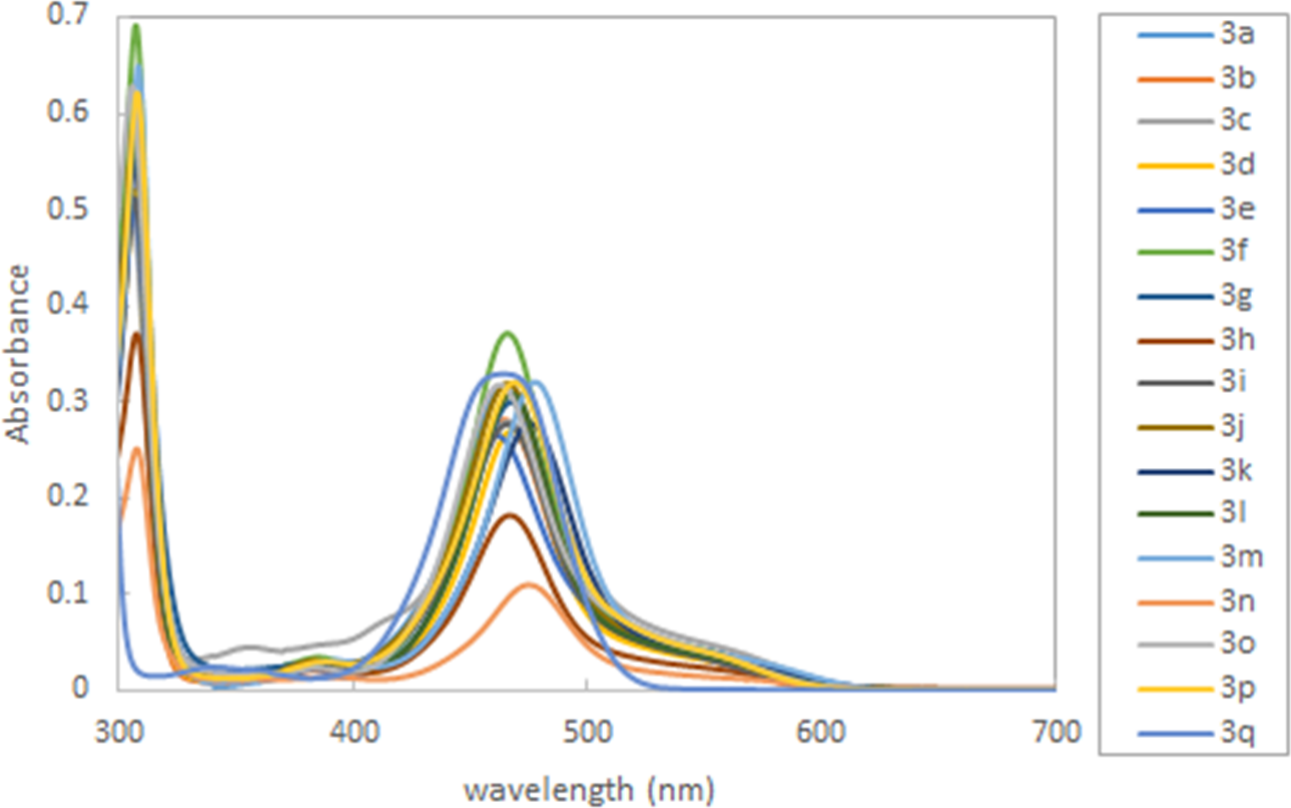
The UV–vis spectra of thioxanthylium salt (0.1 mM) in CH_3_CN.

**Figure 3:**
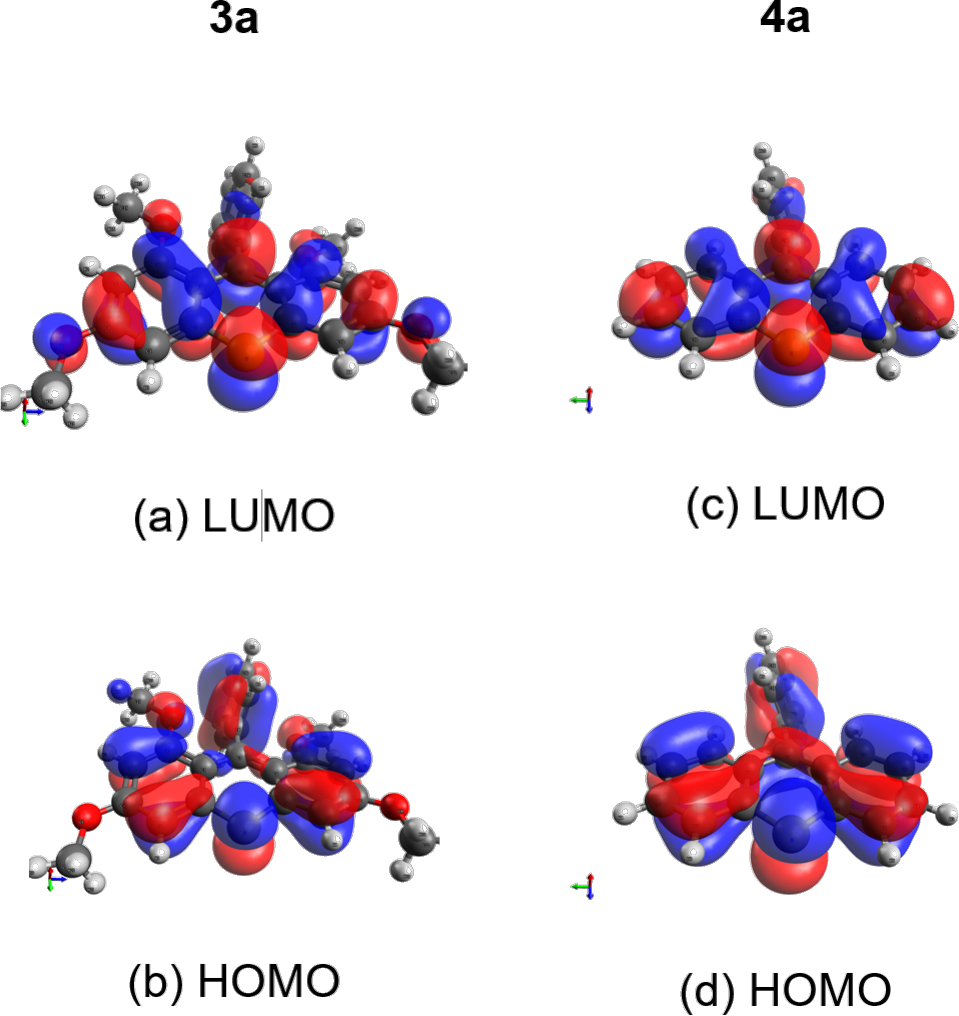
Frontier orbitals of thioxanthylium salts, calculated by DFT at the B3LYP/6-31G(d,p) level of Orca. (a) LUMO localization of **3a** (energy level: −5.745 eV), (b) HOMO localization of **3a** (energy level: −8.842 eV), (c) LUMO localization of **4a** (energy level: −6.812 eV), (d) HOMO localization of **4a** (energy level: −9.788 eV).

**Figure 4:**
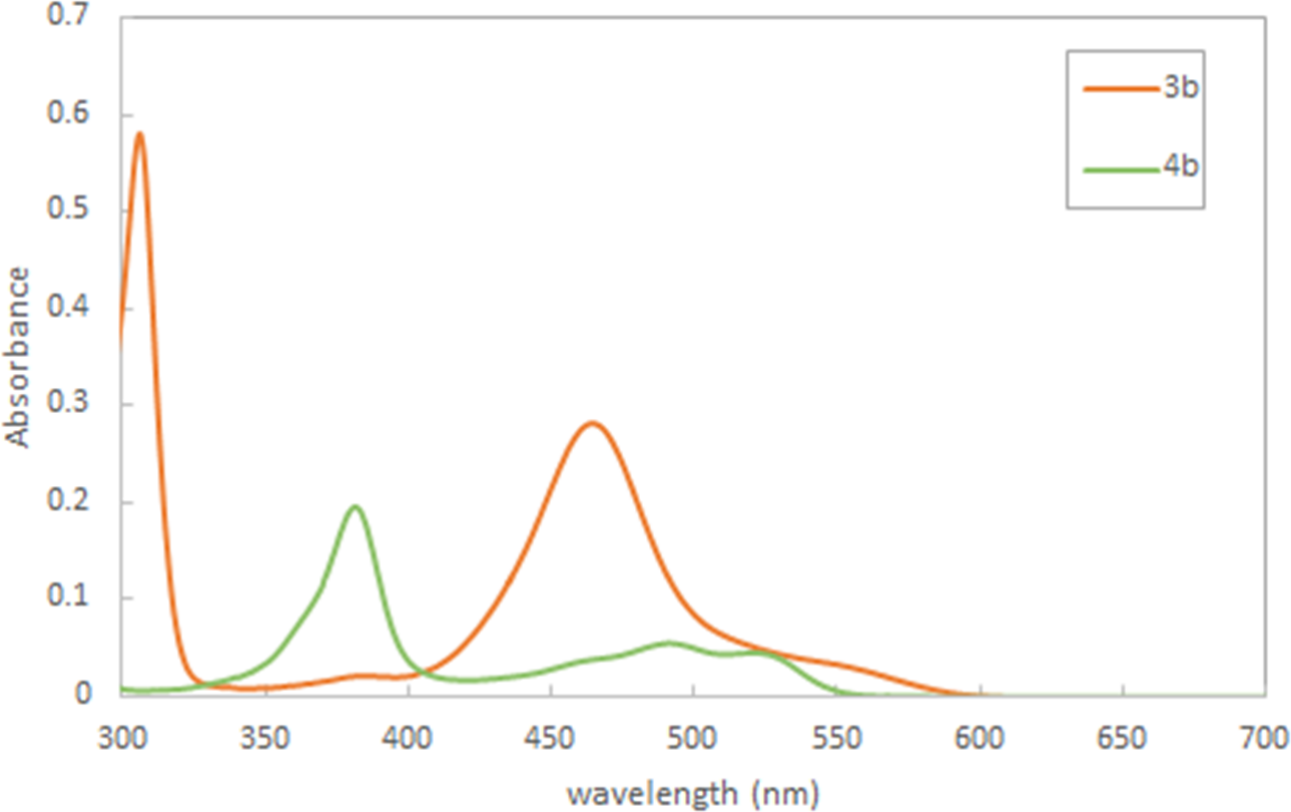
UV–vis spectra of thioxanthylium salts **3b** and **4b** (0.1 mM) in CH_3_CN.

**Figure 5:**
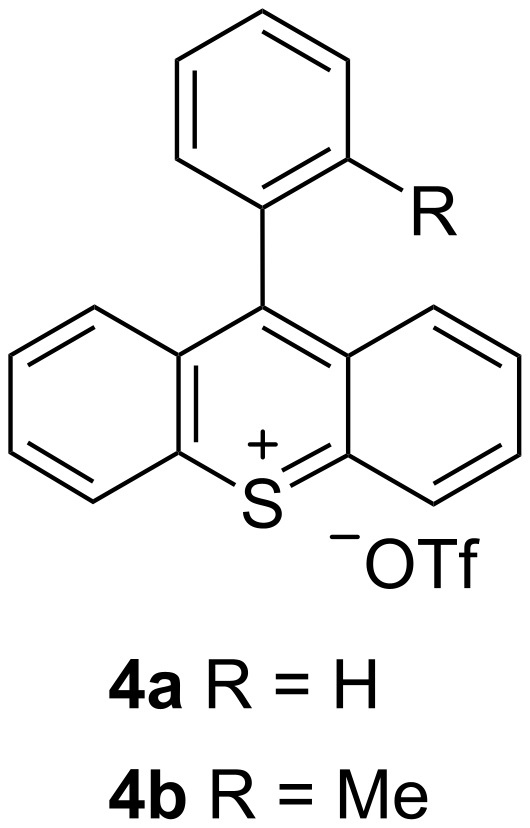
Structure of thioxanthylium salt **4**.

Subsequently, we measured the UV–vis spectra of thioxanthylium salts. As shown in Figure 2, almost all measured UV–vis spectra are nearly identical in spite of different substituents on the benzene ring at the 9-position of the thioxanthylium core. Moreover, when the solvent effects were examined using MeCN, CH_3_NO_2_, DMSO and MeOH, no substantial shifts of the main peak at around 460 nm in the UV–vis absorption spectra were observed [24], indicating that the main absorption of these catalysts would be due to π–π* transition, which is supported by DFT calculations (TD-DFT B3LYP method) (Figure 3a,b). Based on these calculations, it was found that tetramethoxy substituents at the thioxanthylium core lead to an up-shift of both HOMO/LUMO energy levels compared to thioxanthylium salts without methoxy groups (Figure 3c,d). The maximum absorption wavelength of thioxanthylium salt **3b** (λ_max_ = 464 nm) showed a large red shift compared to thioxanthylium salt **4b** (λ_max_ = 383 nm), which has no methoxy groups (Figure 4 and Figure 5).

**Figure 6:**
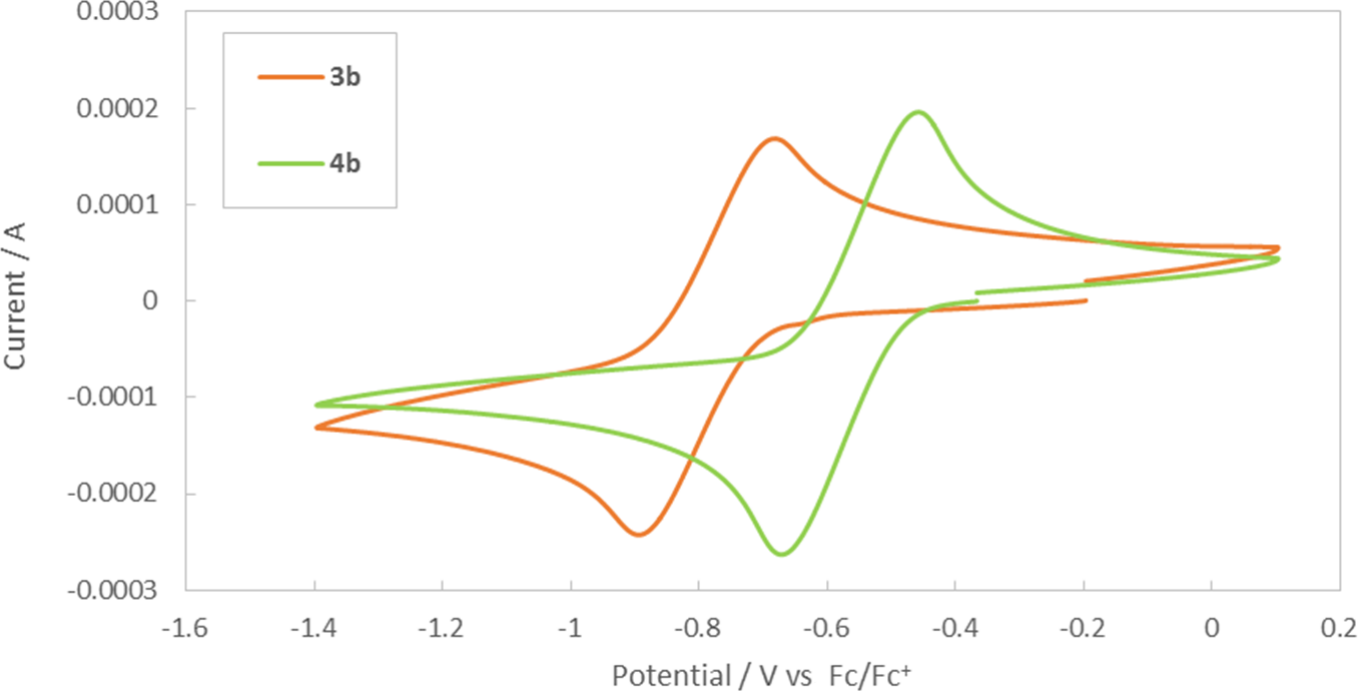
Cyclic voltammograms of thioxanthylium salts **3b** and **4b**.

Finally, we measured the cyclic voltammograms (CV) of thioxanthylium salts **3b** and **4b** (Figure 6). The CV data analysis implies that the reduction potential of **3b** (*E*°’ = −0.79 V vs Fc/Fc^+^) afforded a negative shift compared to **4b** (*E*°’ = −0.56 V vs Fc/Fc^+^). It is obviously indicated that the methoxy groups lower the reduction potential by their strong electron-donating effect.

## Conclusion

We have developed the Friedel–Crafts approach as an efficient method to synthesize oxygen-modified thioxanthylium salts. When the reaction of diaryl sulfide with benzoyl chloride in the presence of TfOH was carried out under reflux conditions in chlorobenzene, the desired thioxanthylium salt was obtained in good yield. A variety of benzoyl chlorides bearing both electron-donating and electron-withdrawing groups can be applied to the reaction. It was found that the main absorption of thioxanthylium salts around 460 nm in UV–vis spectra would be due to π–π* transitions, which was supported by DFT calculations. The present reaction provides a versatile access to functionalized thioxanthylium salts, and therefore constitutes a promising tool for the synthesis of biologically and photochemically active molecules.

## Experimental

### General

Infrared (IR) spectra were recorded on a JASCO FT/IR-4100 spectrophotometer. ^1^H NMR spectra were recorded on a Bruker DRX-300 (300 MHz) spectrometer, a Bruker DRX-500 (500 MHz) spectrometer or a JEOL JNM ECA-500 (500 MHz) spectrometer with tetramethylsilane (TMS) as internal standard. Chemical shifts are reported in ppm from TMS. Data are reported as follows: chemical shift, multiplicity (s = singlet, d = doublet, t = triplet, q = quartet, m = multiplet), coupling constants, integration. ^13^C NMR spectra were recorded on a Bruker DRX-500 (126 MHz) or a JEOL JNM ECA-500 (126 MHz) spectrometer with complete proton decoupling. Chemical shifts are reported in ppm with the solvent resonance as the internal standard (CDCl_3_: δ 77.0). ^19^F NMR spectra were recorded on a JEOL JNM AL-400 (376 MHz) or a JEOL JNM ECA-500 (471 MHz) spectrometer with hexafluorobenzene (C_6_F_6_: δ −164.9 ppm) as internal standard. High-resolution mass spectra (HRMS) were obtained with a Hitachi Nanofrontier LD Spectrometer (ESI/TOF). Elemental analyses of carbon, hydrogen, nitrogen, and sulfur were performed with a CHNOS Elemental Analyzer Vario ELIII Elemental (Elementar Co.). Column chromatography was carried out with Cicareagent silica gel 60 N (spherical, particle size 63–210 mm). Thin-layer chromatography (TLC) was carried out with Merck TLC plates with silica gel 60 F254. Unless otherwise noted, reagents were commercially available and were used without further purification. The UV absorption spectra were measured with a JASCO V-630 spectrometer. Cyclic voltammetry measurements were carried out with a computer-controlled potentiostat Model 660C (ALS Co., Ltd.).

### General procedure for the synthesis of thioxanthylium salt **3**

Analogously as described in [24] thioxanthylium salts **3a**–**q** were prepared according to the following procedure.

A solution of diaryl sulfide **1** (0.25 mmol) and benzoyl chloride **2** (0.75 mmol) in chlorobenzene (5.0 mL) was placed in a 50 mL recovery flask under N_2_. Trifluoromethanesulfonic acid (0.75 mmol) was slowly added to the solution, which was heated to reflux for 1 h. The solution was cooled to room temperature and excess Et_2_O was added to precipitate a solid. After stirred for 1 h, the mixture was filtered. The solid was washed with Et_2_O and dried in vacuo, affording the desired thioxanthylium salt **3**.

### 9-Phenyl-1,3,6,8-tetramethoxythioxanthylium trifluoromethanesulfonate (**3a**)

Red solid (0.1109 g, 82% yield). ^1^H NMR (500 MHz, CDCl_3_) δ 7.51 (d, *J* = 2.2 Hz, 2H), 7.44–7.38 (m, 3H), 7.14–7.10 (m, 2H), 6.51 (d, *J* = 2.5 Hz, 2H), 4.15 (s, 6H), 3.37 (s, 6H); ^13^C NMR (126 MHz, CDCl_3_) δ 168.3. 165.5. 165.2. 147.8. 142.2. 127.2. 127.0. 125.6. 116.9. 101.8. 101.4. 57.7. 56.7; ^19^F NMR (376 MHz, CDCl_3_) δ −81.3; IR (ATR): 1585, 1219, 1143, 1026, 634 cm^−1^; HRMS (ESI^+^) *m*/*z*: [M]^+^ calcd for C_23_H_21_O_4_S, 393.1155; found, 393.1171; anal. calcd for C_24_H_21_F_3_O_7_S_2_: C, 53.13; H, 3.90. found: C, 52.80; H, 4.001.

### For the synthesis of 9-(naphthalene-1-yl)-1,3,6,8-tetramethoxythioxanthylium trifluoromethanesulfonate (**3o**)

To a solution of 1-naphthoic acid (0.1746 g, 1.0 mmol) in dry CH_2_Cl_2_ (2.5 mL) at 0 °C under N_2_ (COCl)_2_ (0.100 mL, 1.2 mmol) was dropwise added. After the addition of a catalytic amount of dry DMF (2 drops), the solution was allowed to warm to room temperature, and stirred at that temperature for 2 h. The reaction mixture was concentrated in vacuo to afford the corresponding crude acid chloride. After the residue was dissolved in chlorobenzene, bis(3,5-dimethoxyphenyl) sulfide **1a** (0.1005 g, 0.33 mmol) and trifluoromethanesulfonic acid (0.087 mL, 0.99 mmol) were added. The reaction temperature was increased to reflux and the solution was stirred for 1 h. The solution was cooled to room temperature and excess Et_2_O was added. After stirring for 1 h, the solution was filtered, and the solid was washed with Et_2_O and dried in vacuo to afford the desired thioxanthylium **3o** (0.1420 g, 73% yield). Red solid. ^1^H NMR (500 MHz, CDCl_3_) δ 7.95 (d, *J* = 8.2 Hz, 1H), 7.88 (d, *J* = 8.2 Hz, 1H), 7.56 (d, *J* = 2.2 Hz, 2H), 7.53–7.45 (m, 2H), 7.36–7.32 (m, 1H), 7.28 (d, *J* = 8.0 Hz, 1H), 6.93 (dd, *J* = 7.1, 1.1 Hz, 1H), 6.43 (d, *J* = 2.2 Hz, 2H), 4.15 (s, 6H), 2.96 (s, 6H); ^13^C NMR (126 MHz, CDCl_3_) δ 168.4, 165.2, 164.7, 147.6, 140.7, 132.1, 132.0, 128.2, 127.5, 126.4, 125.9, 125.0, 124.4, 121.3, 117.6, 102.0, 101.5, 57.7, 56.6; ^19^F NMR (376 MHz, CDCl_3_) δ −81.3; IR (ATR): 1593, 1245, 1148, 1026, 634 cm^−1^; HRMS (ESI^+^) *m*/*z*: [M]^+^ calcd for C_27_H_23_O_4_S, 443.1312; found, 443.1316.

## Supporting Information

File 1 Copies of ^1^H and ^13^C NMR spectra, procedures for the synthesis of diaryl sulfides and thioxanthylium **4**, computational data, absorption spectra, and cyclic voltammetry data.
