# Supplementary material for: Friedel–Crafts approach to the one-pot synthesis of methoxy-substituted thioxanthylium salts
Source: Beilstein J Org Chem. 2019 Sep 5;15:2105–12. doi: 10.3762/bjoc.15.208 (PMC6753671; doi:10.3762/bjoc.15.208)
Supplement: File 1 — Copies of 1H and 13C NMR spectra, procedures for the synthesis of diaryl sulfides and thioxanthylium 4, computational data, absorption spectra, and cyclic voltammetry data. [file Beilstein_J_Org_Chem-15-2105-s001.pdf]

BEILSTEIN JOURNAL OF ORGANIC CHEMISTRY

Supporting Information

for

Friedel–Crafts approach to the one-pot synthesis of methoxy-substituted thioxanthylium salts

Kenta Tanaka, Yuta Tanaka, Mami Kishimoto, Yujiro Hoshino and Kiyoshi Honda

Beilstein J. Org. Chem. **2019,** *15,* 2105–2112. doi:10.3762/bjoc.15.208

Copies of ^{1}H and ^{13}C NMR spectra, procedures for the synthesis of diaryl sulfides and thioxanthylium 4, computational data, absorption spectra, and cyclic voltammetry data

License and Terms: This is a supporting information file under the terms of the Creative Commons Attribution License (http://creativecommons.org/licenses/by/4.0). Please note that the reuse, redistribution and reproduction in particular requires that the authors and source are credited.
The license is subject to the *Beilstein Journal of Organic Chemistry* terms and conditions: (https://www.beilstein-journals.org/bjoc)

Contents

1. Procedure for the synthesis of diaryl sulfide **1**……S2
2. Procedure for the synthesis of thioxanthylium salt **4**……S3
3. Characterization data for thioxanthylium salt **3**……S4
4. Investigation of the Lewis acids……S8
5. Computational data……S9
6. Reference……S12
7. UV–vis spectra of **3** and **4**……S13
8. Cyclic voltammograms of **3** and **4**……S31
9. ^{1}H NMR and ^{13}C NMR data……S33

1. Procedure for the synthesis of diaryl sulfide 1

For the synthesis of bis(dimethoxyphenyl) sulfide

Bis(3,5-dimethoxyphenyl) sulfide and bis(3,4-dimethoxyphenyl) sulfide were prepared according to published methods from 3,5-dimethoxyaniline and 3,4-dimethoxyaniline.[1,2]

Bis(3,5-dimethoxyphenyl) sulfide. White solid (0.7748 g, 63% yield). ^{1}H NMR (300 MHz, $CDCl_3$): δ 6.85 (d, J = 2.3 Hz, 2H), 6.40 (t, J = 2.3 Hz, 1H), 3.76 (s, 6H). The spectroscopic data were agreement with literature values.[1]

Bis(3,4-dimethoxyphenyl) sulfide. White solid (0.6092 g, 60% yield). ^{1}H NMR (500 MHz, $CDCl_3$): δ 6.97-6.86 (m, 4H), 6.86-6.77 (m, 2H), 3.88 (s, 6H), 3.82 (s, 6H). The spectroscopic data were agreement with literature values.[3]

For the synthesis of bis(3,5-diethoxyphenyl) sulfide

1)[1,2,4] 1-Iodo-3,5-dimethoxybenzene were prepared according to published methods from 3,5-dimethoxyaniline.[2] To a solution of 1-iodo-3,5-dimethoxybenzene (0.6824 g, 2.5 mmol) in CH_2Cl_2 (2.5 mL) was placed in a 50 mL recovery flask under N_2. Boron tribromide (10.0 mL, 1.0 M in CH_2Cl_2, 10 mmol) was added slowly at 0 °C. The reaction was warmed to room temperature and stirred overnight. Then, it was cooled to −78 °C, quenched with methanol and concentrated in vacuo. The crude mixture was redissolved in ethyl acetate, washed with H_2O, dried over sodium sulfate, filtered and concentrated in vacuo. The crude product 5-iodobenzene-1,3-diol was used in the subsequent step without further purification.

2)[4] To an oil of 5-iodobenzene-1,3-diol (2.5 mmol) and potassium carbonate (1.3847 g, 10.0 mmol) in DMF (10 mL) were placed in a 100 mL recovery flask under N_2. Bromoethane (0.746 mL, 10.0 mmol) was then added and the reaction heated to 60 °C for 20 h. The reaction was cooled, diluted in ethyl acetate, washed with H_2O, dried over sodium sulfate, filtered and concentrated in vacuo. The crude mixture was purified by column chromatography on silica gel (hexane/ethyl acetate = 20:1) to provide 1,3-diethoxy-5-iodobenzene (0.6014 g, 82 % yield) as a brown oil. ^{1}H NMR (500 MHz, $CDCl_3$): δ 6.83 (d, J = 2.4 Hz, 2H), 6.39 (t, J = 2.0 Hz, 1H), 3.97 (q, J = 6.9 Hz, 4H), 1.38 (t, J = 7.2 Hz, 6H); ^{13}C NMR (126 MHz, $CDCl_3$): δ 160.4, 116.2, 101.4, 94.0, 63.7, 14.7; IR (ATR): 1591, 1434, 1169, 1047, 802, 676 cm^{-1}; LRMS (ESI+) m/z calcd for $C_{10}H_{14}O_2I$ ($[M+H]^+$) : 293.00, found: 293.09.

3)[1] To a mixture of 1,3-diethoxy-5-iodobenzene (0.4857 g, 1.7 mmol), carbon disulfide (0.100 mL, 1.7 mmol), CuI (0.0356 g, 0.17 mmol), and DBU (0.496 mL, 3.3 mmol) in

toluene (3.0 mL) was stirred at reflux under N_2 for 16 h. After H_2O was added, the mixture was extracted with CH_2Cl_2. The organic layer was dried over $MgSO_4$ and concentrated in vacuo. The crude mixture was purified by column chromatography on silica gel (hexane/ethyl acetate = 30:1) to provide bis(3,5-diethoxyphenyl)sulfide (0.0727 g, 39% yield) as a yellow oil.

^{1}H NMR (500 MHz, $CDCl_3$): δ 6.49 (d, *J* = 2.3 Hz, 4H), 6.33 (t, *J* = 2.3 Hz, 2H), 3.96 (q, *J* = 6.9 Hz, 8H), 1.37 (t, *J* = 6.9 Hz, 12H); ^{13}C NMR (126 MHz, $CDCl_3$): δ 160.3, 136.9, 109.3, 100.6, 63.6, 14.7; IR (ATR): 1577, 1430, 1389, 1278, 1166, 1050, 815, 681 cm^{-1}; HRMS (ESI+) m/z calcd for $C_{20}H_{27}O_4S$ ($[M+H]^+$) : 363.1625, found: 363.1639.

2. Procedure for the synthesis of thioxanthylium salt 4

1)[5] To a solution of 2-bromotoluene (0.897 mL, 7.5 mmol) in dioxane (17.0 mL) and THF (28.0 mL) was placed in a 100 mL recovery flask under N_2. *n*-BuLi (5.16 mL, 1.6 M in hexane, 8.26 mmol) was added at -78 °C, and the reaction was stirred for 1 h at −78 °C. Thioxanthone (1.0644 g, 5.0 mmol) in dioxane was added, and the reaction was stirred for 24 h at room temperature. The reaction was quenched with sat. diammonium phosphate and extract with CH_2Cl_2. It was concentrated in vacuo. To the organic layer was added hexane, and the mixture was cooled to −30 °C to give white solid. The mixture was filtered and dried in vacuo, affording the desired 9-(2-methylphenyl)-9-thioxanthenol (0.6863 g, 45% yield) as a white solid. ^{1}H NMR (500 MHz, $CDCl_3$) δ 8.18 (d, *J* = 7.7 Hz, 1H), 7.43-7.36 (m, 3H), 7.32 (t, *J* = 7.4 Hz, 1H), 7.26-7.23 (m, 2H), 7.12-7.04 (m, 5H), 2.51 (d, *J* = 2.9 Hz, 1H), 1.55 (s, 3H). The spectroscopic data were agreement with literature values.[5]

2)[5] To a solution of 9-(2-methylphenyl)-9-thioxanthenol (0.3048 g, 1.0 mmol) in Et_2O (5.0 mL) was placed in a 50 mL recovery flask under N_2. Trifluoromethanesulfonic acid (0.177 mL, 2.0 mmol) was slowly added to precipitate a solid. After stirred for 1 h, the mixture was filtered. The solid was washed with Et_2O and dried in vacuo, affording the desired thioxanthylium salt **4** (0.3473 g, 80% yield). Red solid. ^{1}H NMR (500 MHz, $CDCl_3$): δ 8.91 (d, *J* = 8.6 Hz, 2H), 8.43 (ddd, *J* = 8.3, 6.9, 1.4 Hz, 2H), 8.16 (d, *J* = 8.6 Hz, 2H), 8.07 (ddd, *J* = 8.5, 7.0, 1.2 Hz, 2H), 7.71-7.66 (m, 1H), 7.61-7.53 (m, 2H), 7.33-7.29 (m, 1H), 1.91 (s, 3H); ^{13}C NMR (126 MHz, $CDCl_3$): δ 171.3, 148.1, 137.9, 135.5, 134.5, 133.7, 131.6, 131.0, 131.0, 129.7, 128.8, 128.3, 126.4, 19.7; ^{19}F NMR (471 MHz, $CDCl_3$): δ -81.4; IR (ATR): 1547, 1449, 1373, 1260, 1142, 1029, 736, 634 cm^{-1}; HRMS (ESI+) m/z calcd for $C_{20}H_{15}S$ $[M]^+$: 287.0889, found: 287.0879.

3. Characterization data for thioxanthylium salt 3

9-Phenyl-1,3,6,8-tetramethoxythioxanthylium trifluoromethanesulfonate (**3a**)[1]

Red solid (0.1109 g, 82% yield). ^{1}H NMR (500 MHz, $CDCl_3$): δ 7.51 (d, *J* = 2.2 Hz, 2H), 7.44-7.38 (m, 3H), 7.14-7.10 (m, 2H), 6.51 (d, *J* = 2.5 Hz, 2H), 4.15 (s, 6H), 3.37 (s, 6H); ^{13}C NMR (126 MHz, $CDCl_3$): δ 168.3. 165.5. 165.2. 147.8. 142.2. 127.2. 127.0. 125.6. 116.9. 101.8. 101.4. 57.7. 56.7; ^{19}F NMR (376 MHz, $CDCl_3$): δ -81.3; IR (ATR): 1585, 1219, 1143, 1026, 634 cm^{-1}; HRMS (ESI+) m/z calcd for $C_{23}H_{21}O_4S$ ([M]$^+$): 393.1155, found: 393.1171; EA calcd. for $C_{24}H_{21}F_3O_7S_2$: C, 53.13; H, 3.90. found: C, 52.80; H, 4.001.

9-(2-Methylphenyl)-1,3,6,8-tetramethoxythioxanthylium trifluoromethanesulfonate (**3b**)[6]

Brown solid (0.1274 g, 92% yield). ^{1}H NMR (500 MHz, $CDCl_3$): δ 7.50 (d, *J* = 2.2 Hz, 2H), 7.28 (dd, *J* = 7.3, 1.3 Hz, 1H), 7.26-7.23 (m, 1H), 7.23-7.17 (m, 1H), 6.73 (d, *J* = 7.3 Hz, 1H), 6.54 (d, *J* = 2.2 Hz, 2H), 4.15 (s, 6H), 3.40 (s, 6H), 2.03 (s, 3H); ^{13}C NMR (126 MHz, $CDCl_3$): δ 168.3, 166.0, 165.5, 147.7, 142.1, 134.2, 128.0, 127.5, 125.1, 124.0, 116.7, 102.0, 101.3, 57.7, 56.9, 20.1; ^{19}F NMR (376 MHz, $CDCl_3$): δ -81.3; IR (ATR): 1584, 1220, 1151, 1028, 637 cm^{-1}; HRMS (ESI+) m/z calcd for $C_{24}H_{23}O_4S$ ([M]$^+$) : 407.1312, found: 407.1324.

9-(2-Methoxyphenyl)-1,3,6,8-tetramethoxythioxanthylium trifluoromethanesulfonate (**3c**)

2-Methoxybenzoyl chloride (0.50 mmol), TfOH (0.50 mmol) were used at 120 °C. Red solid (0.1142 g, 80% yield). ^{1}H NMR (500 MHz, $CDCl_3$): δ 7.49 (d, *J* = 2.2 Hz, 2H), 7.38 (td, *J* = 8.0, 1.7 Hz, 1H), 6.98 (t, *J* = 7.5 Hz, 1H), 6.95 (d, *J* = 8.2 Hz, 1H), 6.72 (dd, *J* = 7.3, 1.6 Hz, 1H), 6.53 (d, *J* = 2.5 Hz, 2H), 4.15 (s, 6H), 3.73 (s, 3H), 3.43 (s, 6H); ^{13}C NMR (126 MHz, $CDCl_3$): δ 168.1, 165.4, 163.6, 156.4, 147.8, 132.2, 129.0, 125.2, 120.3, 117.4, 108.5, 101.7, 101.4, 57.7, 56.8, 55.7; ^{19}F NMR (376 MHz, $CDCl_3$): δ -81.3; IR (ATR): 1585, 1217, 1149, 1026, 636 cm^{-1}; HRMS (ESI+) m/z calcd for $C_{24}H_{23}O_5S$ ([M]$^+$): 423.1261, found: 423.1256.

9-(2-Trifluoromethylphenyl)-1,3,6,8-tetramethoxythioxanthylium trifluoromethanesulfonate (**3d**)[1]

Brown solid (0.0906 g, 59% yield). ^{1}H NMR (500 MHz, $CDCl_3$): δ 7.77 (d, *J* = 7.0 Hz, 1H), 7.61-7.54 (m, 2H), 7.53 (d, *J* = 2.5 Hz, 2H), 6.98 (dd, *J* = 8.2, 3.5 Hz, 1H), 6.53 (d, *J* = 2.5 Hz, 2H), 4.15 (s, 6H), 3.39 (s, 6H); ^{13}C NMR (126 MHz, $CDCl_3$): δ 168.5, 164.9, 162.4, 147.4, 140.1 (q, *J* = 2.8 Hz), 131.1, 127.7, 127.5 (q, *J* = 30.9 Hz), 126.0, 125.0 (q, *J* = 4.6

Hz), 124.3 (q, *J* = 274.0 Hz), 120.9 (q, *J* = 320.5 Hz, OTf⁻), 116.4, 102.1, 101.2, 57.7, 56.9; ^{19}F NMR (376 MHz, $CDCl_3$): δ -63.0, -81.3; IR (ATR): 1585, 1237, 1222, 1029, 637 cm^{-1}; HRMS (ESI+) m/z calcd for $C_{24}H_{20}O_4F_3S$ ($[M]^+$): 461.1029, found: 461.1037; EA calcd. for $C_{25}H_{20}F_6O_7S_2$: C, 49.18; H, 3.30; S, 10.50. found: C, 48.97; H, 3.280; S, 10.30.

9-(4-Methoxyphenyl)-1,3,6,8-tetramethoxythioxanthylium trifluoromethanesulfonate (**3e**)[1]

4-Methoxybenzoyl chloride (0.50 mmol), TfOH (0.50 mmol) were used. Brown solid (0.1158 g, 81% yield). ^{1}H NMR (500 MHz, $CDCl_3$): δ 7.40 (d, *J* = 2.2 Hz, 2H), 7.03 (d, *J* = 8.0 Hz, 2H), 6.97 (d, *J* = 8.2 Hz, 2H), 6.54 (d, *J* = 2.2 Hz, 2H), 4.14 (s, 6H), 3.91 (s, 3H), 3.45 (s, 6H); ^{13}C NMR (126 MHz, $CDCl_3$): δ 168.3, 165.9, 165.8, 159.2, 147.3, 134.4, 127.2, 117.3, 112.5, 101.7, 101.2, 57.6, 56.8, 55.5; ^{19}F NMR (376 MHz, $CDCl_3$): δ -81.3; IR (ATR): 1587, 1219, 1146, 1027, 635 cm^{-1}; HRMS (ESI+) m/z calcd for $C_{24}H_{23}O_5S$ ($[M]^+$): 423.1261, found: 423.1253. EA calcd. for $C_{24}H_{21}F_3O_7S_2$: C, 52.44; H, 4.05. found: C, 51.75; H, 4.285.

9-(4-Trifluoromethylphenyl)-1,3,6,8-tetramethoxythioxanthylium trifluoromethanesulfonate (**3f**)

4-(Trifluoromethyl)benzoyl chloride (0.50 mmol), TfOH (0.50 mmol) were used. Brown solid (0.0990 g, 65% yield). ^{1}H NMR (500 MHz, $CDCl_3$): δ 7.72 (d, *J* = 2.0 Hz, 2H), 7.60-7.59 (m, 2H), 7.32 (d, *J* = 2.0 Hz, 2H), 6.51 (d, *J* = 0.6 Hz, 2H), 4.17 (s, 6H), 3.35 (s, 6H); ^{13}C NMR (126 MHz, $CDCl_3$): δ 168.5, 165.0, 162.7, 147.8, 146.1, 129.3 (q, *J* = 32.5 Hz), 126.2, 124.1 (q, *J* = 272.8 Hz), 123.8 (q, *J* = 3.5 Hz), 120.8 (q, *J* = 320.9 Hz, OTf⁻), 116.3, 102.1, 101.5, 57.8, 56.6; ^{19}F NMR (376 MHz, $CDCl_3$): δ -65.6, -81.3; IR (ATR): 1592, 1403, 1219, 1027, 840, 636 cm^{-1}; HRMS (ESI+) m/z calcd for $C_{24}H_{20}O_4F_3S$ ($[M]^+$): 461.1029, found: 461.1041.

9-(4-Nitrophenyl)-1,3,6,8-tetramethoxythioxanthylium trifluoromethanesulfonate (**3g**)

The reaction was carried out for 20 h. Black solid (0.1261 g, 86% yield). ^{1}H NMR (500 MHz, $CDCl_3$): δ 8.33 (d, *J* = 8.8 Hz, 2H), 7.55 (d, *J* = 2.5 Hz, 2H), 7.42-7.40 (m, 2H), 6.53 (d, *J* = 2.2 Hz, 2H), 4.15 (s, 6H), 3.38 (s, 6H); ^{13}C NMR (126 MHz, $CDCl_3$): δ 168.7,164.6, 161.3, 149.4, 148.0, 146.8, 127.1, 122.2, 116.1, 102.3, 101.7, 57.8, 56.7; ^{19}F NMR (376 MHz, $CDCl_3$): δ -81.4; IR (ATR): 1584, 1219, 1150, 1027, 837, 634 cm^{-1}; HRMS (ESI+) m/z calcd for $C_{23}H_{20}NO_6S$ ($[M]^+$): 438.1006, found: 438.0991.

9-(4-Cyanophenyl)-1,3,6,8-tetramethoxythioxanthylium trifluoromethanesulfonate (**3h**)
Black solid (0.1359 g, 96% yield). ^{1}H NMR (500 MHz, $CDCl_3$): δ 7.76 (d, *J* = 2.0 Hz, 2H), 7.58 (d, *J* = 0.5 Hz, 2H), 7.34 (d, *J* = 2.3 Hz, 2H), 6.53-6.51 (m, 2H), 4.17 (s, 6H), 3.39 (s, 6H); ^{13}C NMR (126 MHz, $CDCl_3$): δ 168.6, 164.9, 161.8, 147.5, 147.3, 130.7, 126.7, 120.6 (q, *J* = 320.9 Hz, OTf^-), 118.7, 115.9, 110.6, 102.2, 101.4, 57.7, 56.7; ^{19}F NMR (376 MHz, $CDCl_3$): δ -81.4; IR (ATR): 1584, 1402, 1213, 1154, 1028, 823, 636 cm^{-1}; HRMS (ESI+) m/z calcd for $C_{24}H_{20}NO_4S$ ($[M]^+$): 418.1108, found: 418.1098.

9-(4-Fluorophenyl)-1,3,6,8-tetramethoxythioxanthylium trifluoromethanesulfonate (**3i**)
Brown solid (0.0970 g, 69% yield). ^{1}H NMR (500 MHz, $CDCl_3$): δ 7.55 (d, *J* = 2.3 Hz, 2H), 7.18-7.09 (m, 4H), 6.52 (d, *J*=2.3 Hz, 2H), 4.16 (s, 6H), 3.43 (s, 6H); ^{13}C NMR (126 MHz, $CDCl_3$): δ 168.4, 165.3, 164.1, 162.0 (d, *J* = 247.6 Hz), 147.7, 138.0, 127.5 (d, *J* = 7.2 Hz), 116.9, 114.1 (d, *J* = 21.7 Hz), 101.8, 101.4, 57.7, 56.7; ^{19}F NMR (471 MHz, $CDCl_3$): δ -81.3, -117.4; IR (ATR): 1585, 1415, 1220, 1153, 1027, 836, 636, 573 cm^{-1}; HRMS (ESI+) m/z calcd for $C_{23}H_{20}O_4FS$ ($[M]^+$): 411.1061, found: 411.1064.

9-(4-Chlorophenyl)-1,3,6,8-tetramethoxythioxanthylium trifluoromethanesulfonate (**3j**)
Brown solid (0.0951 g, 66% yield). ^{1}H NMR (500 MHz, $CDCl_3$): δ 7.54 (d, *J* = 2.3 Hz, 2H), 7.46-7.40 (m, 2H), 7.13-7.06 (m, 2H), 6.52 (d, *J* = 2.9 Hz, 2H), 4.16 (s, 6H), 3.43 (s, 6H); ^{13}C NMR (126 MHz, $CDCl_3$): δ 168.5, 165.3, 163.6, 147.5, 140.6, 133.1, 127.2, 127.1, 116.6, 102.0, 101.3, 57.7, 56.7; ^{19}F NMR (376 MHz, $CDCl_3$): δ -81.3; IR (ATR): 1588, 1424, 1219, 1027, 850, 635, 571 cm^{-1}; HRMS (ESI+) m/z calcd for $C_{23}H_{20}O_4SCl$ ($[M]^+$): 427.0765, found: 427.0767.

9-(2,6-Difluorophenyl)-1,3,6,8-tetramethoxythioxanthylium trifluoromethanesulfonate (**3k**)
Brown solid (0.1177 g, 81% yield). ^{1}H NMR (500 MHz, $CDCl_3$): δ 7.61 (d, *J* = 2.2 Hz, 2H), 7.45-7.40 (m, 1H), 7.02-6.98 (m, 2H), 6.60 (d, *J* = 2.5 Hz, 2H), 4.18 (s, 6H), 3.54 (s, 6H); ^{13}C NMR (126 MHz, $CDCl_3$): δ 168.5, 164.9, 158.5 (dd, *J* = 252.0, 11.0 Hz), 151.7, 147.9, 129.9 (t, *J* = 9.6 Hz), 120.9 (q, *J* = 320.8 Hz, OTf^-), 118.9 (t, *J* = 20.2 Hz), 116.9, 110.3 (dd, *J* = 19.3, 5.5 Hz), 102.2, 101.5, 57.9, 57.4; ^{19}F NMR (376 MHz, $CDCl_3$): δ -81.3, -116.6; IR (ATR): 1593,1245, 1148, 1026, 634 cm^{-1}; HRMS (ESI+) m/z calcd for $C_{23}H_{19}O_4F_2S$ ($[M]^+$): 429.0967, found: 429.0966.

9-(3,5-Difluorophenyl)-1,3,6,8-tetramethoxythioxanthylium trifluoromethanesulfonate (**3l**)

The reaction was carried out for 2 h. Black Solid (0.0793 g, 55% yield). ^{1}H NMR (500 MHz, $CDCl_3$): δ 7.60-7.53 (m, 2H), 6.91 (t, J = 8.6 Hz, 1H), 6.75 (d, J = 6.7 Hz, 2H), 6.56 (s, 2H), 4.17 (s, 6H), 3.51 (s, 6H); ^{13}C NMR (126 MHz, $CDCl_3$): δ 168.6, 165.0, 162.3 (dd, J = 250.0, 13.2 Hz), 160.8, 147.7, 145.1 (t, J = 10.8 Hz), 116.0, 109.3 (dd, J = 21.7, 6.0 Hz), 102.5 (t, J = 25.3 Hz), 102.2, 101.4, 57.8, 57.1; ^{19}F NMR (376 MHz, $CDCl_3$): δ -81.3, -113.9; IR (ATR): 1586, 1404, 1218, 1141, 1027, 850, 635 cm^{-1}; HRMS (ESI+) m/z calcd for $C_{23}H_{19}O_4F_2S$ ([M]$^+$): 429.0967, found: 429.0968.

9-(2,4,6-Trichlorophenyl)-1,3,6,8-tetramethoxythioxanthylium trifluoromethanesulfonate (**3m**)[1]

2,4,6-Trichlorobenzoyl chloride (0.50 mmol), TfOH (0.50 mmol) were used at 120 °C. Brown solid (0.0417 g, 33% yield). ^{1}H NMR (500 MHz, $CDCl_3$): δ 7.68 (d, J = 2.2 Hz, 2H), 7.45 (s, 2H), 6.60 (d, J = 2.2 Hz, 2H), 4.19 (s, 6H), 3.58 (s, 6H); ^{13}C NMR (126 MHz, $CDCl_3$): δ 168.5, 164.5, 156.9, 148.3, 139.0, 134.1, 132.0, 126.8, 115.8, 102.6, 101.5, 58.0, 57.7; ^{19}F NMR (376 MHz, $CDCl_3$): δ -81.3; IR (ATR): 1586, 1222, 1154, 1029, 636 cm^{-1}; HRMS (ESI+) m/z calcd for $C_{23}H_{18}O_4SCl_3$ ([M]$^+$): 494.9986, found: 464.9999; EA calcd. for $C_{24}H_{18}Cl_3F_3O_7S_2$: C, 44.63; H, 2.81. found: C, 44.58; H, 2.975.

9-(2,3,4,5-Tetrachlorophenyl)-1,3,6,8-tetramethoxythioxanthylium trifluoromethanesulfonate (**3n**)

Brown solid (0.1259 g, 74% yield). ^{1}H NMR (500 MHz, $CDCl_3$) δ 7.60 (d, J = 2.5 Hz, 2H), 7.10 (s, 1H), 6.61 (d, J = 2.2 Hz, 2H), 4.18 (s, 6H), 3.57 (s, 6H); ^{13}C NMR (126 MHz, $CDCl_3$) δ 168.6, 164.2, 156.9, 148.5, 141.9, 132.1, 131.9, 131.3, 130.2, 125.0, 115.9, 102.5, 102.0, 58.0, 57.4; ^{19}F NMR (376 MHz, $CDCl_3$): δ -81.3; IR (ATR): 1594, 1248, 1231, 1161, 1024, 638 cm^{-1}; HRMS (ESI+) m/z calcd for $C_{23}H_{17}O_4SCl_4$ ([M]$^+$): 528.9596, found: 528.9614.

9-(2-Methylphenyl)-1,3,6,8-tetraethoxythioxanthylium trifluoromethanesulfonate (**3p**)

Brown solid (0.0975 g, 51% yield). ^{1}H NMR (500 MHz, $CDCl_3$): δ 7.50 (d, J = 2.3 Hz, 2H), 7.40-7.31 (m, 1H), 7.21-7.16 (m, 2H), 6.79 (d, J = 7.5 Hz, 1H), 6.48 (d, J = 2.3 Hz, 2H), 4.42 (q, J = 7.3 Hz, 4H), 3.80-3.69 (m, 2H), 3.69-3.59 (m, 2H), 2.04 (s, 3H), 1.52 (t, J = 6.9 Hz, 6H), 0.79 (t, J = 7.2 Hz, 6H); ^{13}C NMR (126 MHz, $CDCl_3$): δ 167.5, 165.3, 164.9, 147.5, 142.2, 134.7, 128.9, 127.9, 125.4, 124.7, 116.5, 101.9, 101.6, 66.5, 65.4, 20.2, 14.3,

13.2; ^{19}F NMR (376 MHz, $CDCl_3$): δ -81.3; IR (ATR): 1586, 1442, 1214, 1028, 824, 635 cm^{-1}; HRMS (ESI+) m/z calcd for $C_{28}H_{31}O_4S$ ([M]$^+$): 463.1938, found: 463.1937.

9-(2-Methylphenyl)-2,3,6,7-tetramethoxythioxanthylium trifluoromethanesulfonate **(3q)**

Brown solid (0.0937 g, 67% yield). ^{1}H NMR (500 MHz, $CDCl_3$): δ 8.23 (s, 2H), 7.69-7.61 (m, 1H), 7.58-7.52 (m, 2H), 7.23 (d, *J* = 6.9 Hz, 1H), 6.94 (s, 2H), 4.29 (s, 6H), 3.74 (s, 6H), 1.93 (s, 3H); ^{13}C NMR (126 MHz, $CDCl_3$): δ 159.8, 157.5, 152.1, 143.3, 135.3, 134.9, 131.2, 130.6, 128.2, 126.9, 124.8, 109.7, 107.8, 58.2, 56.1, 19.4; ^{19}F NMR (376 MHz, $CDCl_3$): δ -81.3; IR (ATR): 1608, 1506, 1426, 1222, 1029, 748, 636, 571 cm^{-1}; HRMS (ESI+) m/z calcd for $C_{24}H_{23}O_4S$ ([M]$^+$): 407.1312, found: 407.1315.

9-(naphthalene-1-yl)-1,3,6,8-tetramethoxythioxanthylium trifluoromethanesulfonate **(3o)**

Red solid (0.1420 g, 73% yield). ^{1}H NMR (500 MHz, $CDCl_3$): δ 7.95 (d, *J* = 8.2 Hz, 1H), 7.88 (d, *J* = 8.2 Hz, 1H), 7.56 (d, *J* = 2.2 Hz, 2H), 7.53-7.45 (m, 2H), 7.36-7.32 (m, 1H), 7.28 (d, *J* = 8.0 Hz, 1H), 6.93 (dd, *J* = 7.1, 1.1 Hz, 1H), 6.43 (d, *J* = 2.2 Hz, 2H), 4.15 (s, 6H), 2.96 (s, 6H); ^{13}C NMR (126 MHz, $CDCl_3$): δ 168.4, 165.2, 164.7, 147.6, 140.7, 132.1, 132.0, 128.2, 127.5, 126.4, 125.9, 125.0, 124.4, 121.3, 117.6, 102.0, 101.5, 57.7, 56.6; ^{19}F NMR (376 MHz, $CDCl_3$): δ -81.3; IR (ATR): 1593, 1245, 1148, 1026, 634 cm^{-1}; HRMS (ESI+) m/z calcd for $C_{27}H_{23}O_4S$ [M]$^+$: 443.1312, found: 443.1316.

4. Investigation of the Lewis acids

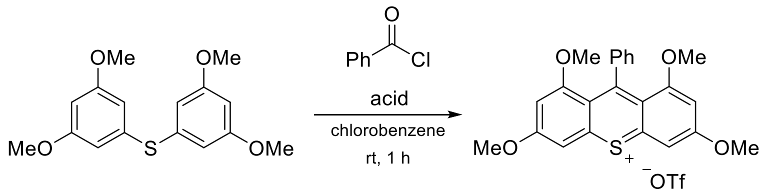

Entry	acid	Yield (%)
1	$AlCl_3$	0
2	BF_3•OEt_2	0
3	$Sc(OTf)_3$	0

When we investigated the Lewis acids such as $AlCl_3$, BF_3・OEt_2 and $Sc(OTf)_3$ the reaction did not proceed, suggesting that TfOH is the best acid in the reaction system.

5. Computational data

Structure optimizations and frequency analyses of cations **3a** and **4a** were carried out at the B3LYP level of density functional theory (DFT) with the 6-31G(d,p) basis set on the ORCA program package.[7]

9-Phenyl-1,3,6,8-tetramethoxythioxanthylium cation

atom type	x	y	z
C	0.098289	0.212300	-1.259567
C	-0.048430	1.637149	-1.378192
C	-0.186286	2.306630	-2.592329
C	-0.119796	1.574514	-3.781309
C	0.151294	0.193490	-3.741241
C	0.302373	-0.475146	-2.540210
C	-0.000172	-0.460670	0.000080
C	-0.098675	0.211989	1.259956
C	0.048120	1.636846	1.378826
S	-0.000139	2.693604	0.000423
C	-0.302955	-0.475562	2.540575
C	-0.151790	0.192919	3.741679
C	0.119541	1.573877	3.781928
C	0.186088	2.306135	2.593051
C	0.000200	-1.961032	-0.000366
C	1.156660	-2.662899	0.361318
C	1.156940	-4.057529	0.352482
C	0.001028	-4.759099	-0.001674
C	-1.155263	-4.057882	-0.355283
C	-1.155778	-2.663242	-0.362910
O	-0.667038	-1.760043	2.494520
O	0.665809	-1.759825	-2.494120
O	-0.258582	2.093560	-5.004712
O	0.258367	2.092753	5.005398
C	-0.865079	-2.501778	3.702304
C	0.863396	-2.501759	-3.701854
C	0.512693	3.495491	5.160825

C	-0.512713	3.496352	-5.159959
H	-0.324325	3.381135	-2.600956
H	0.266673	-0.315514	-4.688717
H	-0.267378	-0.316162	4.689094
H	0.324224	3.380627	2.601827
H	2.056687	-2.120362	0.638908
H	2.060648	-4.596207	0.624789
H	0.001345	-5.845936	-0.002171
H	-2.058632	-4.596838	-0.628159
H	-2.056077	-2.120977	-0.640150
H	-1.149078	-3.501501	3.375429
H	-1.669374	-2.062156	4.302908
H	0.059428	-2.550035	4.288427
H	1.146890	-3.501606	-3.374917
H	1.667892	-2.062607	-4.302532
H	-0.061165	-2.549581	-4.287927
H	0.604156	3.655430	6.234780
H	-0.321808	4.088171	4.769363
H	1.446889	3.779068	4.663824
H	-0.604154	3.656443	-6.233893
H	0.321868	4.088869	-4.768422
H	-1.446870	3.779995	-4.662921

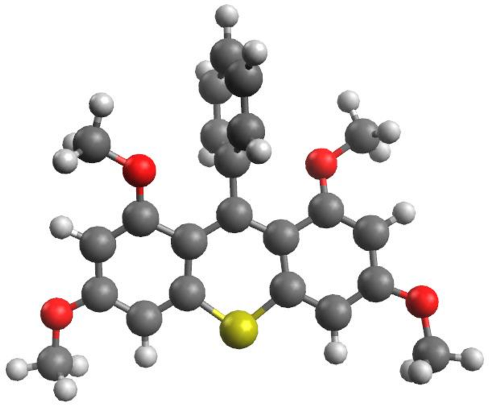

Total Energy: -1587.13327102 hartree

Imaginary Frequencies: 0

9-Phenylthioxanthylium cation

atom type	x	y	z
C	0.012766	-1.262464	0.390821
C	-0.013433	-1.376506	1.819830
C	-0.027469	-2.637633	2.445485
C	0.004556	-3.788733	1.680883
C	0.070917	-3.710651	0.273252
C	0.081039	-2.485682	-0.351445
C	0.002161	-0.001927	-0.283219
C	-0.007121	1.253571	0.400184
C	0.021920	1.356969	1.829933
S	0.005312	-0.013698	2.885260
C	-0.076875	2.482279	-0.332817
C	-0.065479	3.702563	0.300969
C	0.003715	3.770176	1.709005
C	0.037236	2.613398	2.464933
C	0.000773	0.003640	-1.772225
C	1.117101	0.483200	-2.481241
C	1.116962	0.473661	-3.875672
C	-0.001963	0.014655	-4.575388
C	-1.119498	-0.449897	-3.877117
C	-1.116914	-0.470398	-2.482804
H	-0.149117	2.437877	-1.411587
H	0.151000	-2.433155	-1.429995
H	-0.004855	-4.757405	2.171275
H	0.014137	4.735158	2.206593
H	-0.056602	-2.700380	3.529520
H	0.124918	-4.619712	-0.316437
H	-0.120675	4.615985	-0.281847
H	0.068557	2.668066	3.549349
H	1.988954	0.841032	-1.941344
H	1.990884	0.827526	-4.414024
H	-0.003056	0.019015	-5.661318
H	-1.994473	-0.799519	-4.416525

H	-1.987748	-0.832423	-1.944042

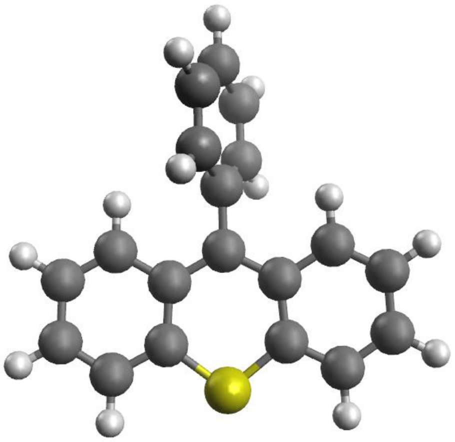

Total Energy: -1129.58253481 hartree

Imaginary Frequencies: 0

6. Reference

1. Tanaka, K.; Kishimoto, M.; Sukekawa, M.; Hoshino, Y.; Honda, K. *Tetrahedron Lett.* **2018**, *59*, 3361–3364.

2. Mao, W.; Wang, T.; Zeng, H.; Wang, Z.; Chen, J.; Shen, J. *Bioorg. Med. Chem. Lett.* **2009**, *19*, 4570-4573.

3. Trosien, S.; Bottger, P.; Waldvogel, S. R. *Org. Lett.* **2014**, *16*, 402-405.

4. Szymaniak, A. A.; Zhang, C.; Coombs, J. R.; Morken, J. P. *ACS Catal.* **2018**, *8*, 2897-2901.

5. Erabi, T.; Asahara, M.; Miyamoto, M.; Goto, K.; Wada, M. *Bull. Chem. Soc. Jpn.* **2002**, *75*, 1325-1332.

6. Tanaka, K.; Omata, D.; Asada, Y.; Hoshino, Y.; Honda, K. *J. Org. Chem.* **2019**, DOI: 10.1021/acs.joc.9b01156.

7. For the ORCA program system; a) Neese, F.; *Wiley Interdiscip. Rev.: Comput. Mol. Sci.* **2012**, *2*, 73-78. b) Neese, F. *Wiley Interdiscip. Rev.: Comput. Mol. Sci.* **2017**, *8*, e1327.

7. UV–vis spectrum of 3 and 4

UV-vis spectra of **3** and **4** (0.1 mM) in CH_3CN.

3a

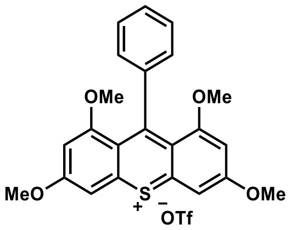

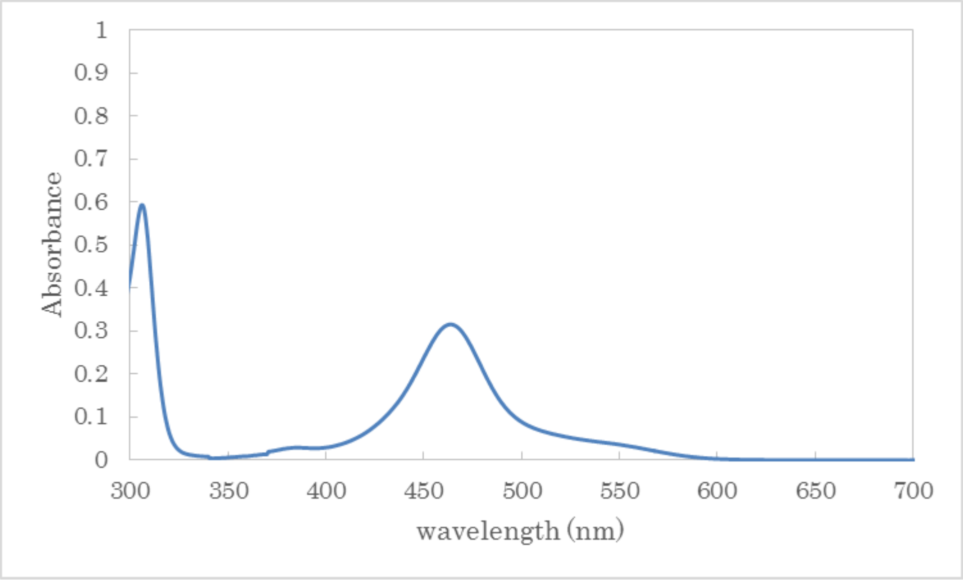

3b

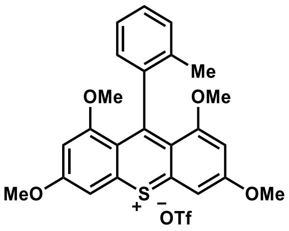
Me
OMe
OMe
MeO
OMe
S
OTf

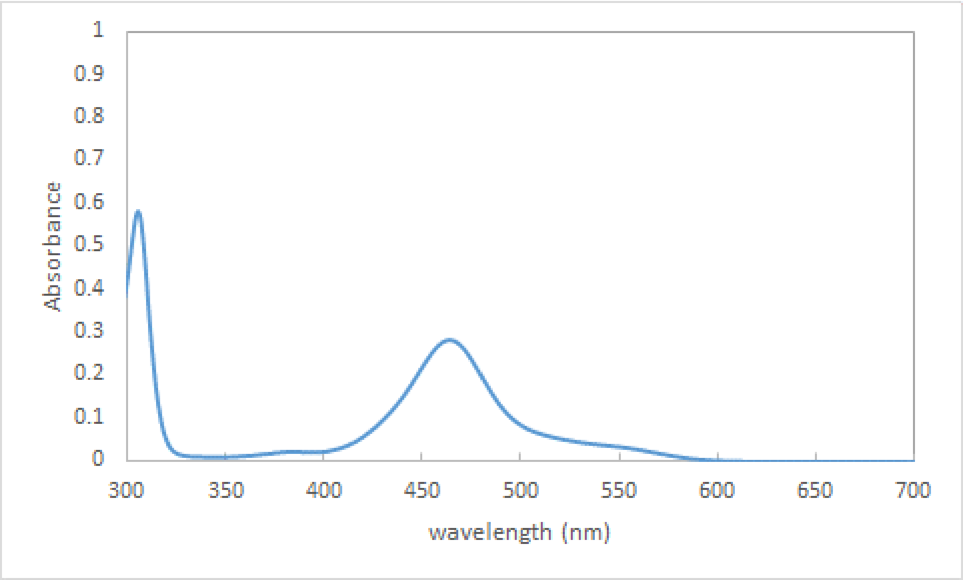
Absorbance
1
0.9
0.8
0.7
0.6
0.5
0.4
0.3
0.2
0.1
0
300
350
400
450
500
550
600
650
700
wavelength (nm)

3c

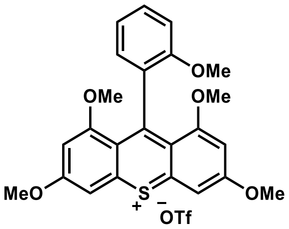
OMe
OMe
OMe
MeO
S
OTf
OMe

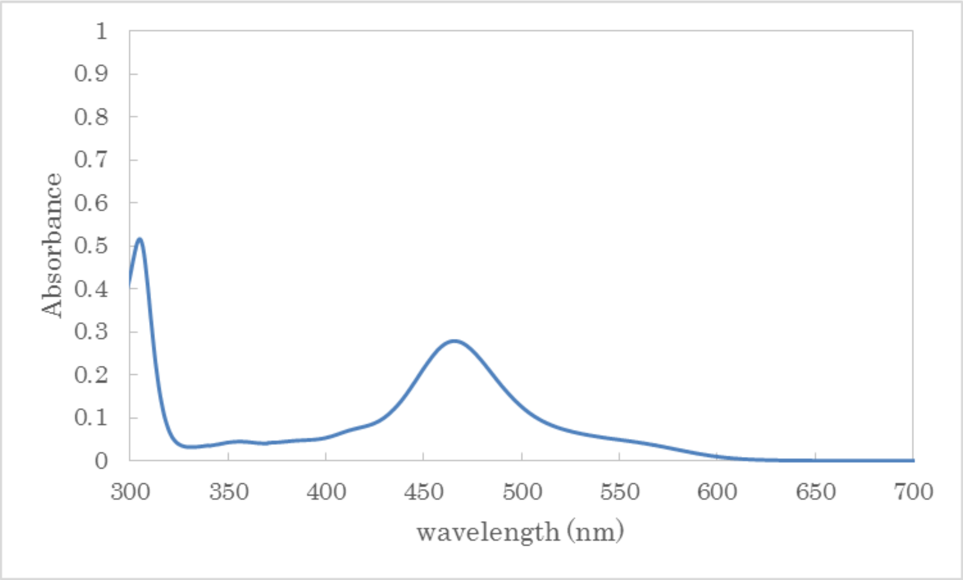
Absorbance
1
0.9
0.8
0.7
0.6
0.5
0.4
0.3
0.2
0.1
0
300
350
400
450
500
550
600
650
700
wavelength (nm)

3d

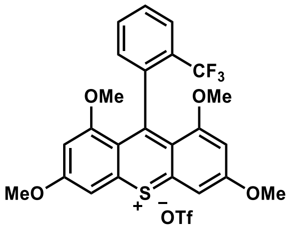

CF_3
OMe
OMe
MeO
S
+
−
OTf
OMe

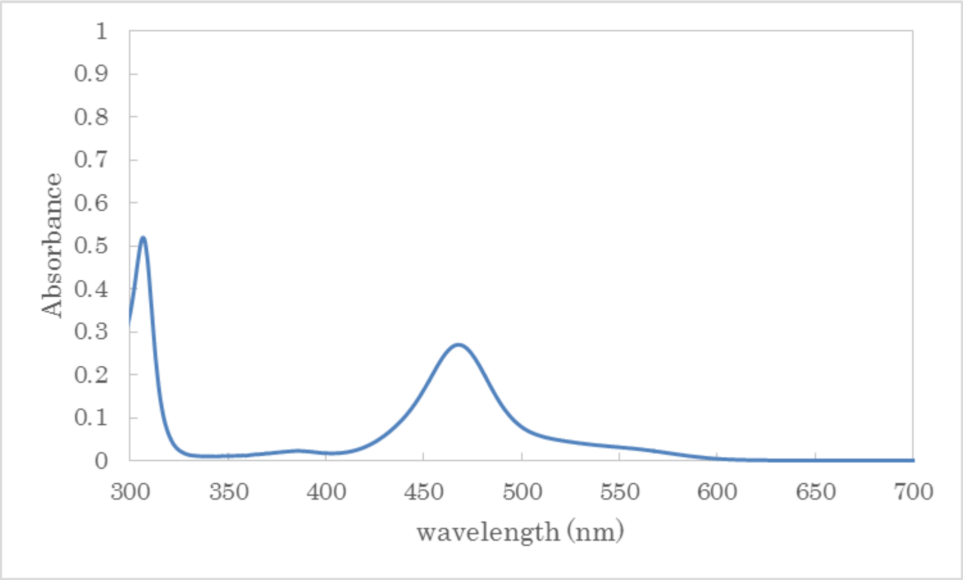

Absorbance
1
0.9
0.8
0.7
0.6
0.5
0.4
0.3
0.2
0.1
0
300
350
400
450
500
550
600
650
700
wavelength (nm)

3e

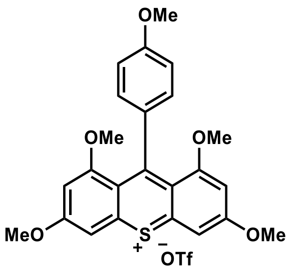
OMe
OMe
OMe
MeO
OMe
S
OTf

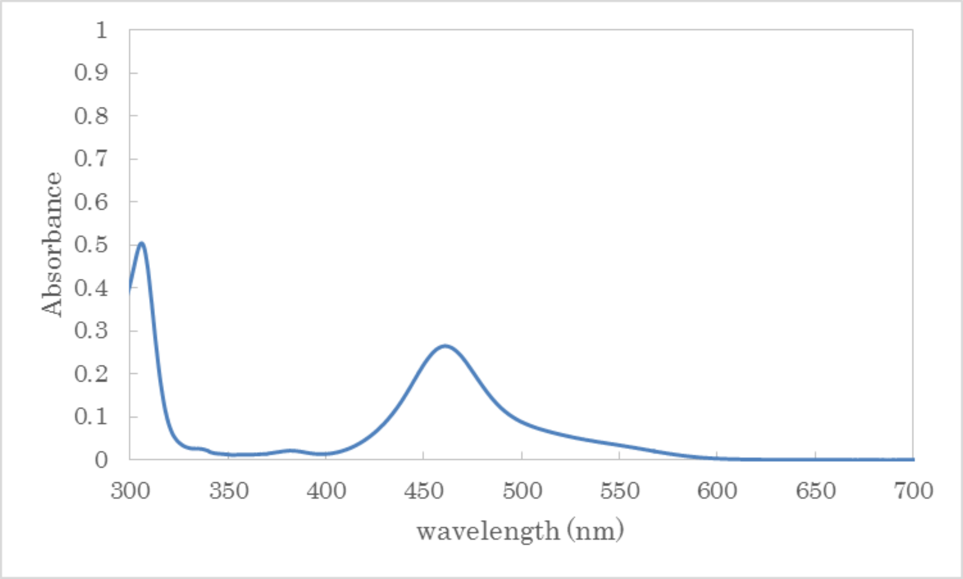
Absorbance
1
0.9
0.8
0.7
0.6
0.5
0.4
0.3
0.2
0.1
0
300
350
400
450
500
550
600
650
700
wavelength (nm)

3f

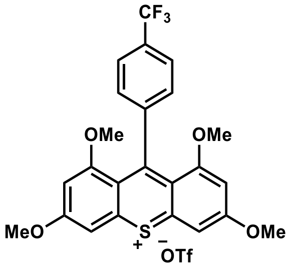
CF3
OMe
OMe
MeO
OMe
S
+
–
OTf

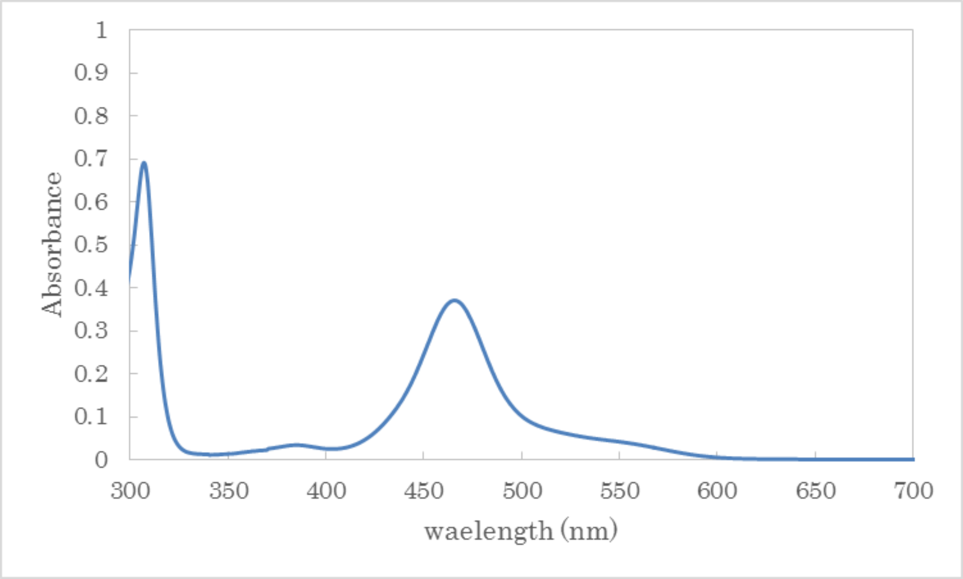
Absorbance
1
0.9
0.8
0.7
0.6
0.5
0.4
0.3
0.2
0.1
0
300
350
400
450
500
550
600
650
700
waelength (nm)

3g

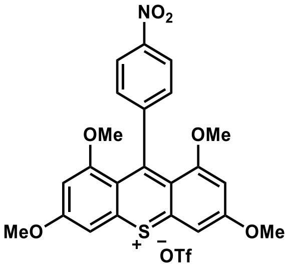
NO2
OMe
OMe
MeO
OMe
S
+
−
OTf

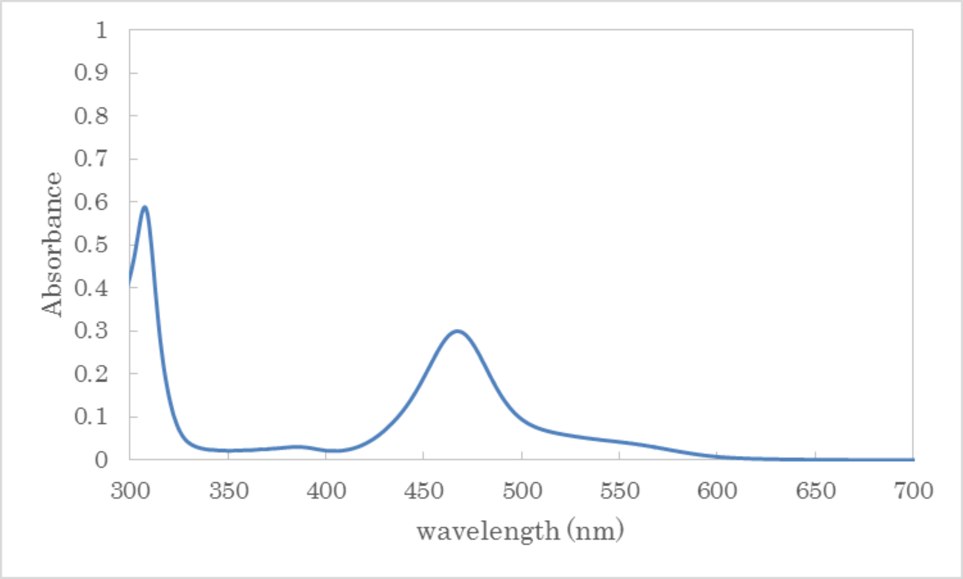
Absorbance
1
0.9
0.8
0.7
0.6
0.5
0.4
0.3
0.2
0.1
0
300
350
400
450
500
550
600
650
700
wavelength (nm)

3h

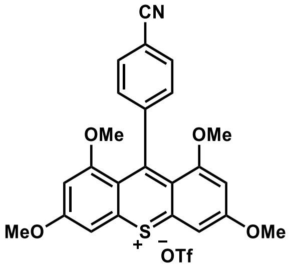
CN
OMe
OMe
MeO
OMe
S
+
−
OTf

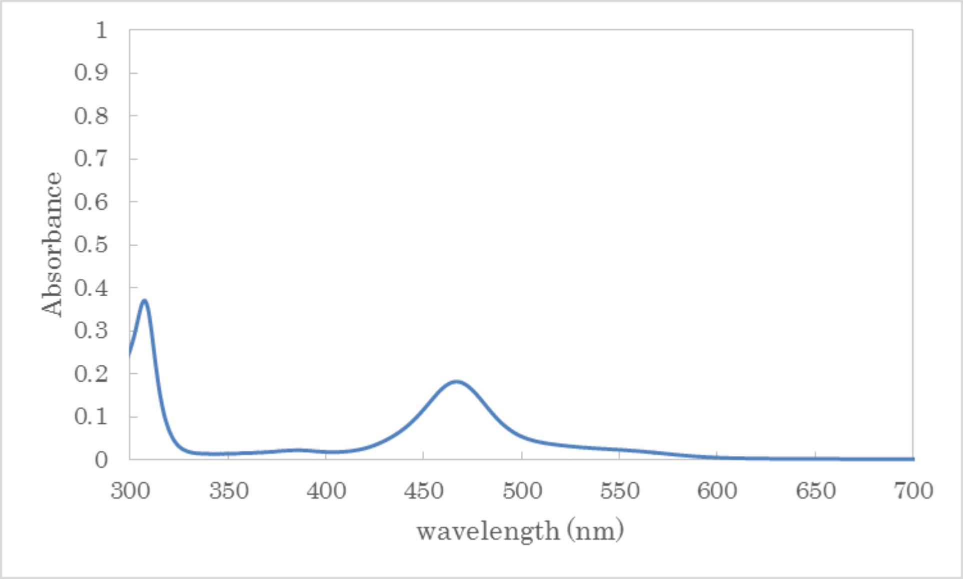
Absorbance
1
0.9
0.8
0.7
0.6
0.5
0.4
0.3
0.2
0.1
0
300
350
400
450
500
550
600
650
700
wavelength (nm)

3i

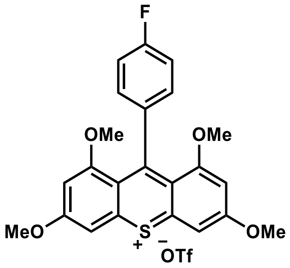
F
OMe
OMe
MeO
OMe
S
+
−
OTf

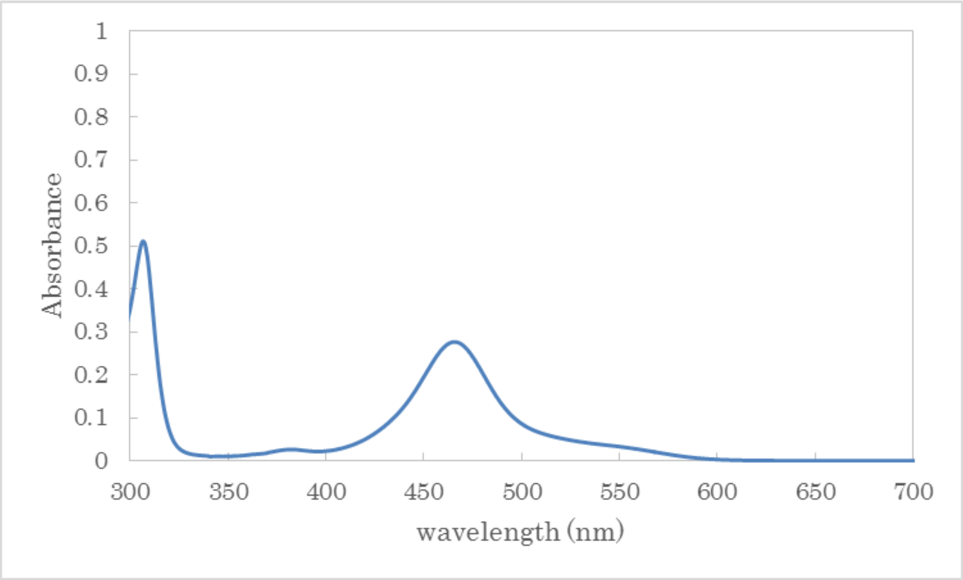
Absorbance
1
0.9
0.8
0.7
0.6
0.5
0.4
0.3
0.2
0.1
0
300
350
400
450
500
550
600
650
700
wavelength (nm)

3j

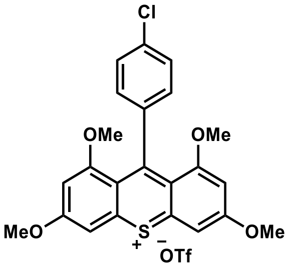
Cl
OMe
OMe
MeO
OMe
S
+
−
OTf

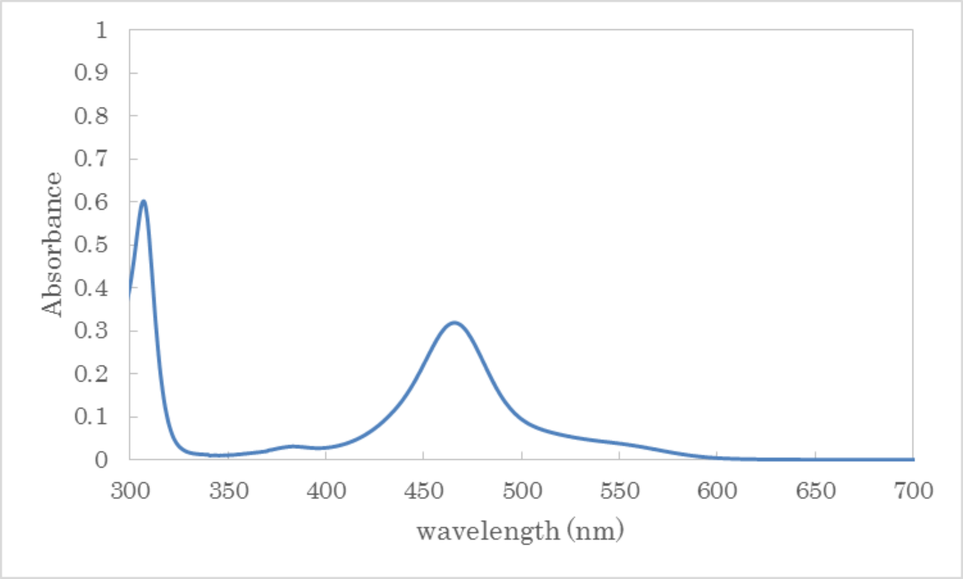
1
0.9
0.8
0.7
0.6
0.5
0.4
0.3
0.2
0.1
0
Absorbance
300
350
400
450
500
550
600
650
700
wavelength (nm)

3k

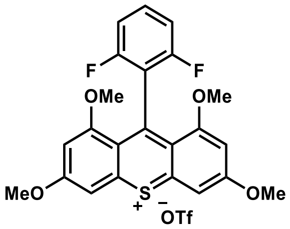
F
F
OMe
OMe
MeO
S
OTf
OMe

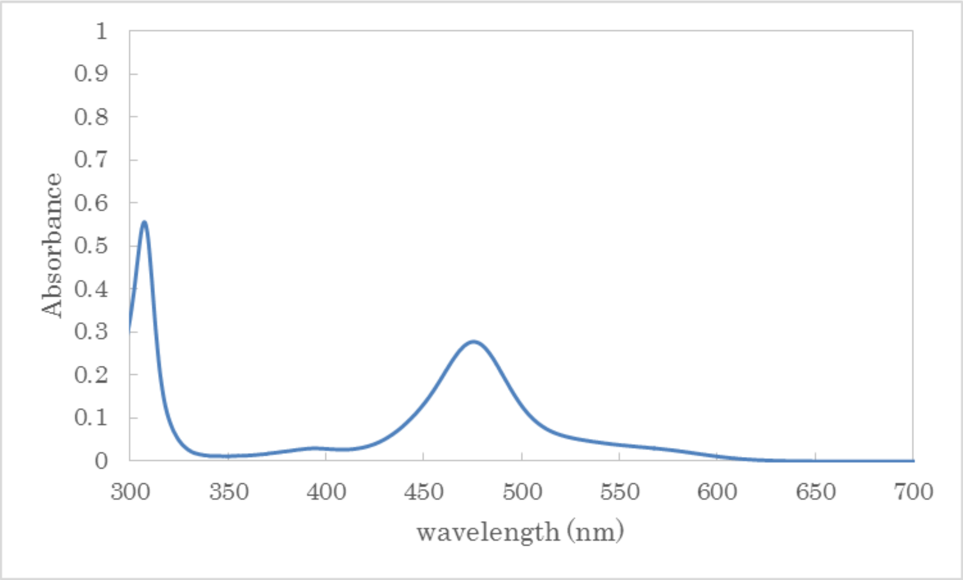
Absorbance
0
0.1
0.2
0.3
0.4
0.5
0.6
0.7
0.8
0.9
1
300
350
400
450
500
550
600
650
700
wavelength (nm)

31

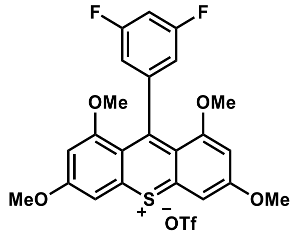
F
F
OMe
OMe
MeO
OMe
S
+
−
OTf

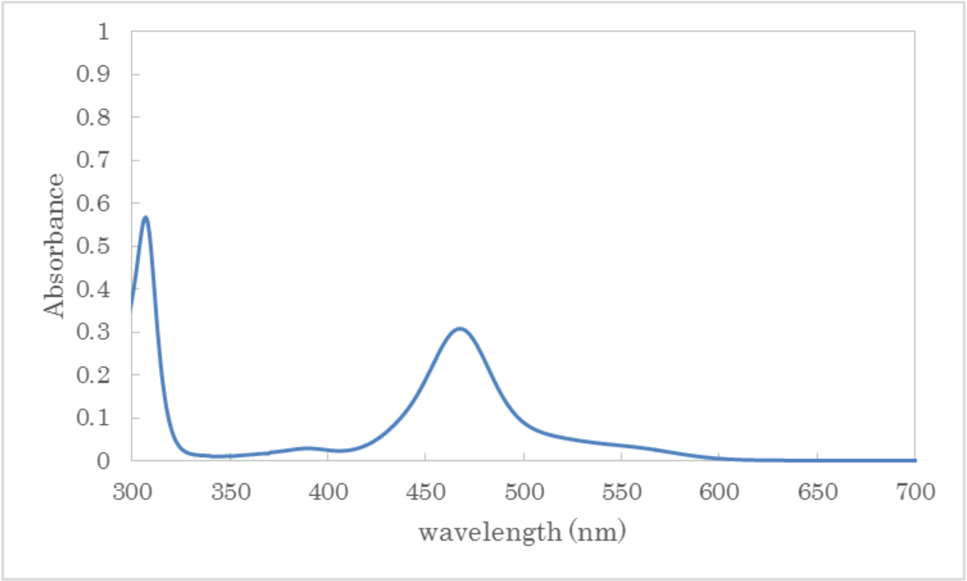
1
0.9
0.8
0.7
0.6
0.5
0.4
0.3
0.2
0.1
0
Absorbance
300
350
400
450
500
550
600
650
700
wavelength (nm)

3m

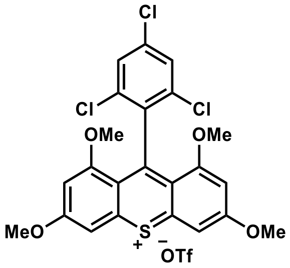
Cl
Cl
Cl
OMe
OMe
MeO
OMe
S
+
−
OTf

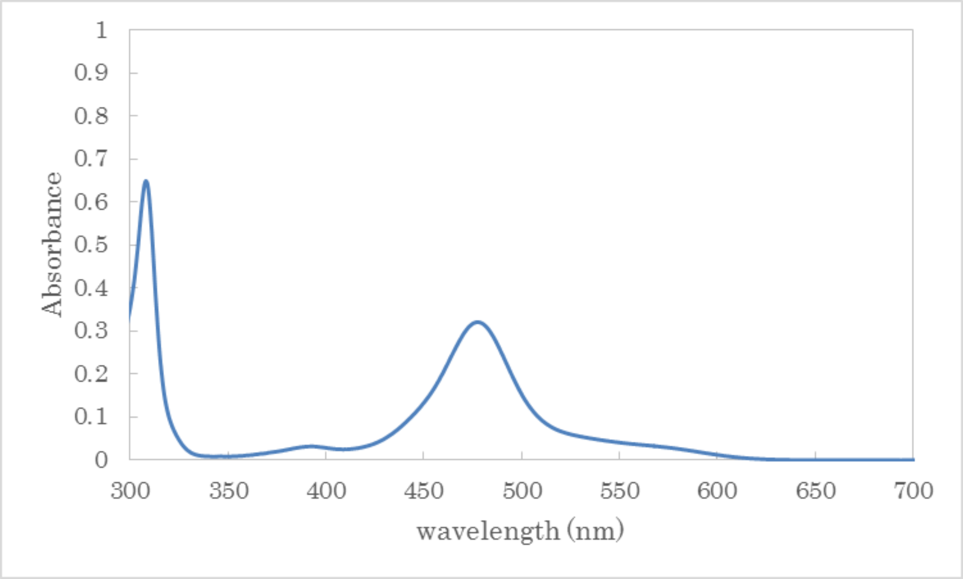
1
0.9
0.8
0.7
0.6
0.5
0.4
0.3
0.2
0.1
0
Absorbance
300
350
400
450
500
550
600
650
700
wavelength (nm)

3n

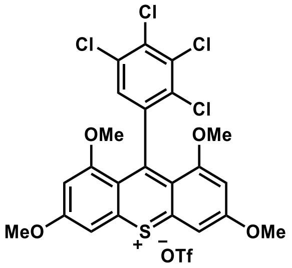
Cl
Cl
Cl
Cl
OMe
OMe
MeO
OMe
S
+
−
OTf

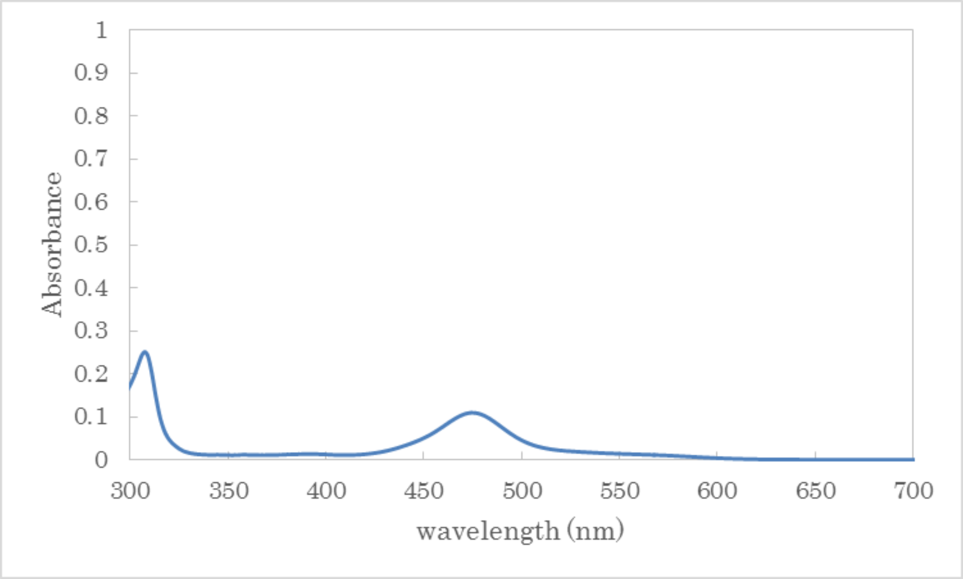
Absorbance
1
0.9
0.8
0.7
0.6
0.5
0.4
0.3
0.2
0.1
0
300
350
400
450
500
550
600
650
700
wavelength (nm)

3o

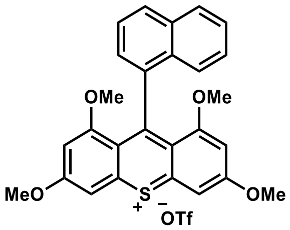
OMe
OMe
MeO
S
OTf
OMe

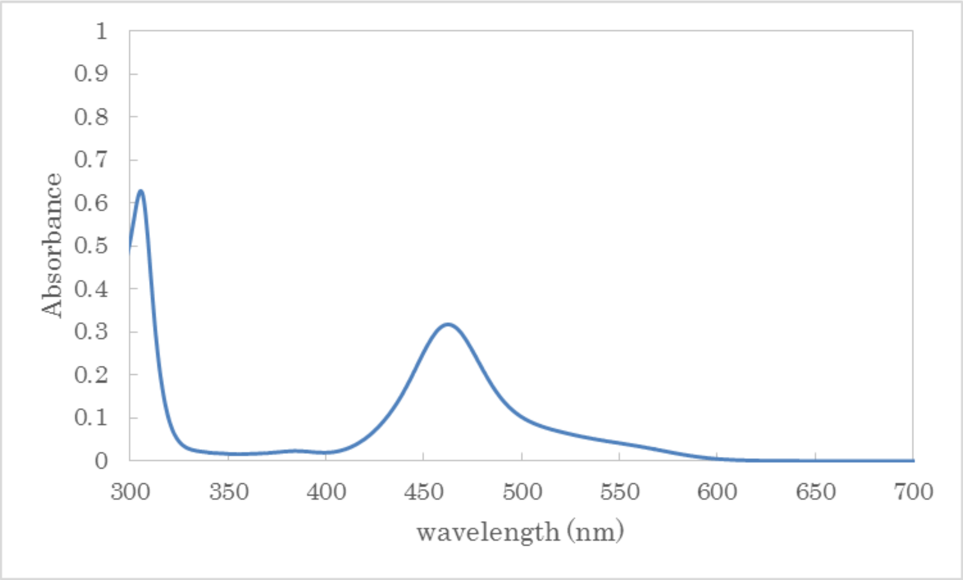
1
0.9
0.8
0.7
0.6
0.5
0.4
0.3
0.2
0.1
0
Absorbance
300
350
400
450
500
550
600
650
700
wavelength (nm)

3p

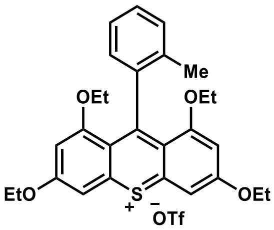
Me
OEt
OEt
EtO
S
OTf
OEt

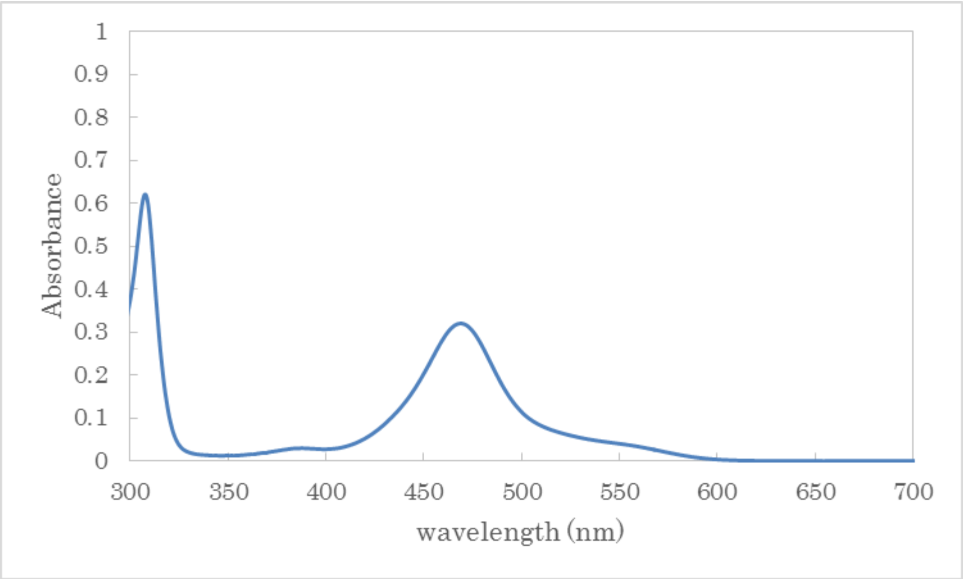
1
0.9
0.8
0.7
0.6
0.5
0.4
0.3
0.2
0.1
0
Absorbance
300
350
400
450
500
550
600
650
700
wavelength (nm)

3q

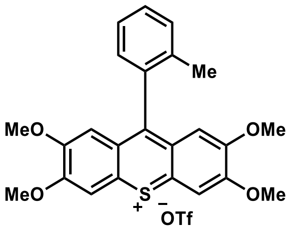
Me
MeO
OMe
MeO
OMe
S
+
−
OTf

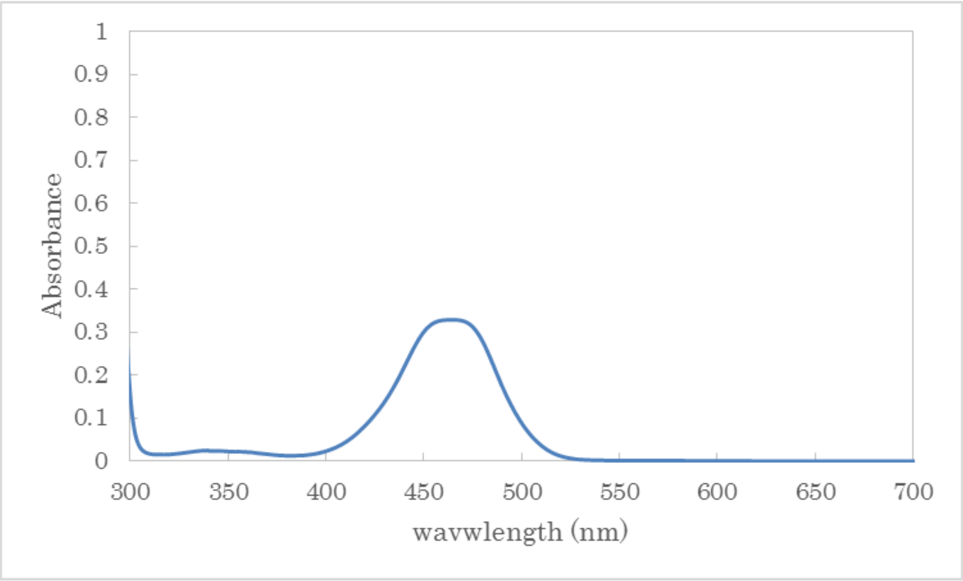
1
0.9
0.8
0.7
0.6
0.5
0.4
0.3
0.2
0.1
0
Absorbance
300
350
400
450
500
550
600
650
700
wavwlength (nm)

4b

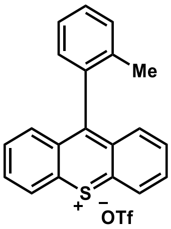
Me
S
+
−
OTf

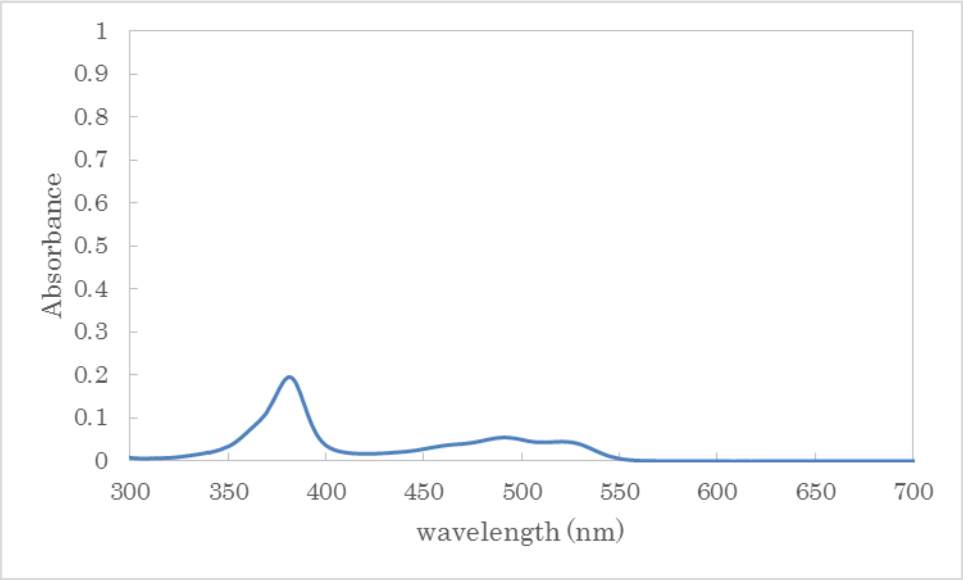
Absorbance
wavelength (nm)
0
0.1
0.2
0.3
0.4
0.5
0.6
0.7
0.8
0.9
1
300
350
400
450
500
550
600
650
700

8. Cyclic voltammograms of 3 and 4

Samples for electrochemical measurements were prepared with 10 mL of a 0.01 M tetra-*n*-butylammonium tetrafluoroborate solution in dry CH_3CN and 0.01 mmol of thioxanthylium salt. Cyclic voltammetry measurements were carried out with a computer-controlled potentiostat Model 660C (ALS Co., Ltd.). Cyclic Voltammetry was recorded using an undivided cell equipped with a working electrode (Pt disk electrode, φ 3 mm), a counter electrode (Pt wire), and a reference electrode (Ag wire). The ferrocene / ferrocenium couple (Fc/Fc^+) was also measured in the same electrochemical system, and the electrode potential was reported as values referred to the apparent standard potential of the system. A scan rate was used 1.0 V/s.

3b

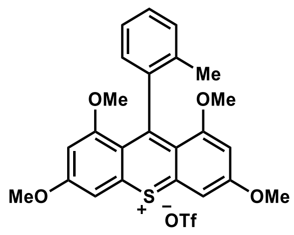

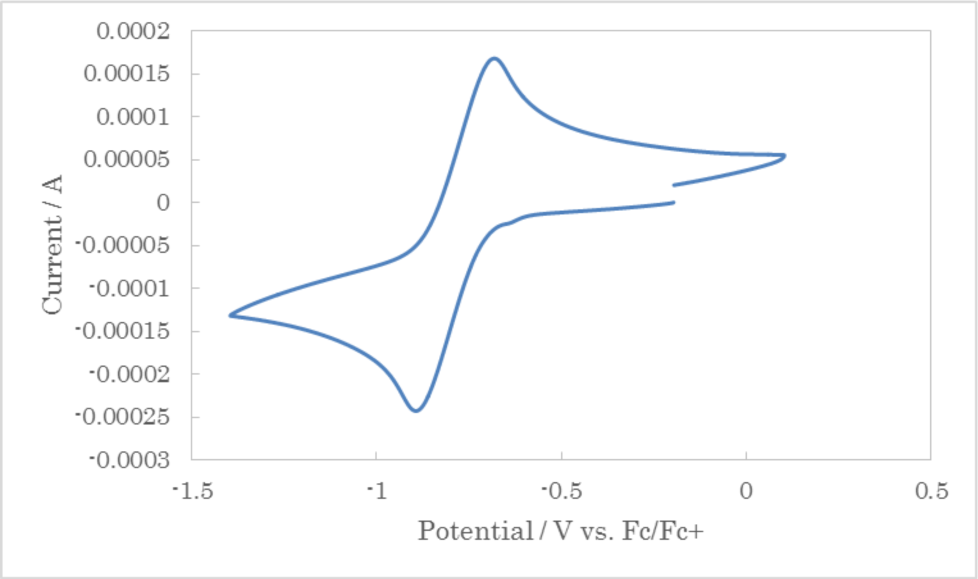

$E_0' = -0.79$ V *vs.* Fc/Fc^+

4b

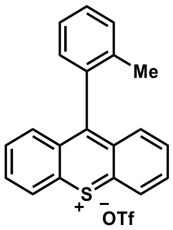

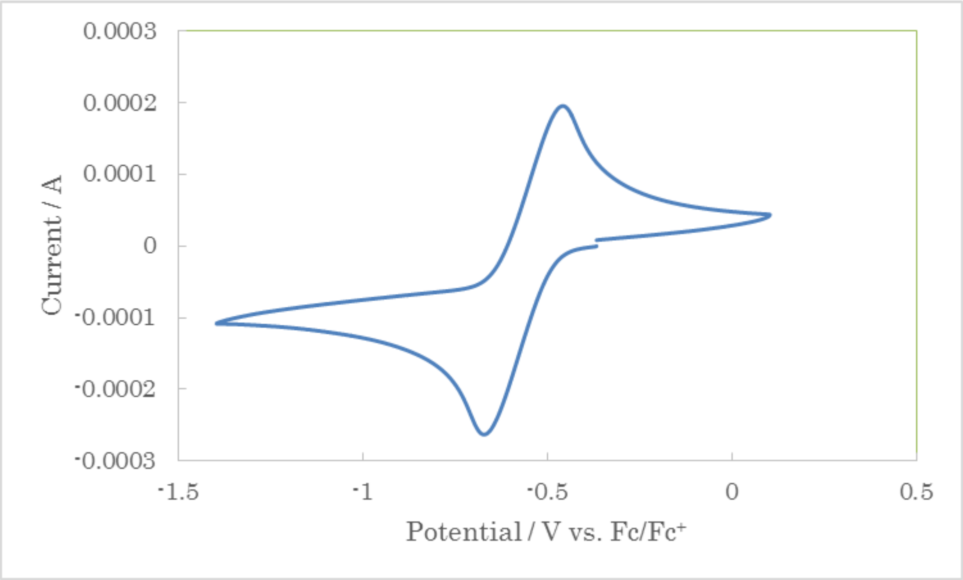

$E_0' = -0.56\ \mathrm{V}\ vs.\ \mathrm{Fc/Fc^+}$

9. ¹H NMR and ¹³C NMR spectrum data

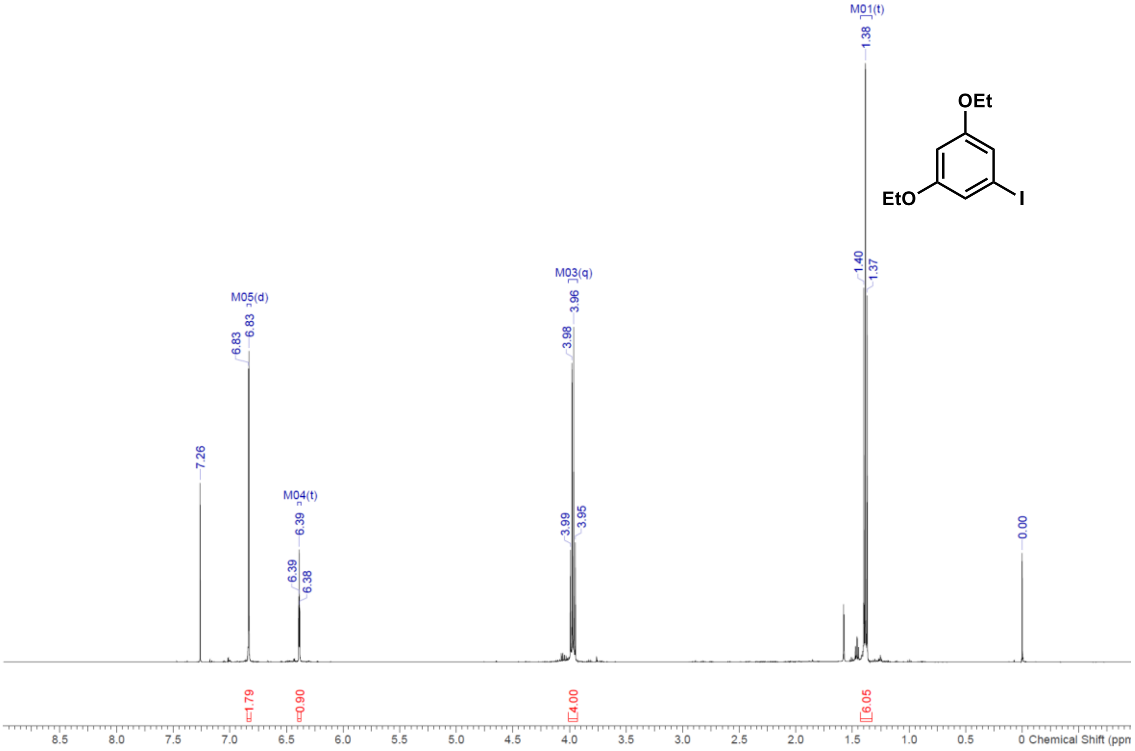

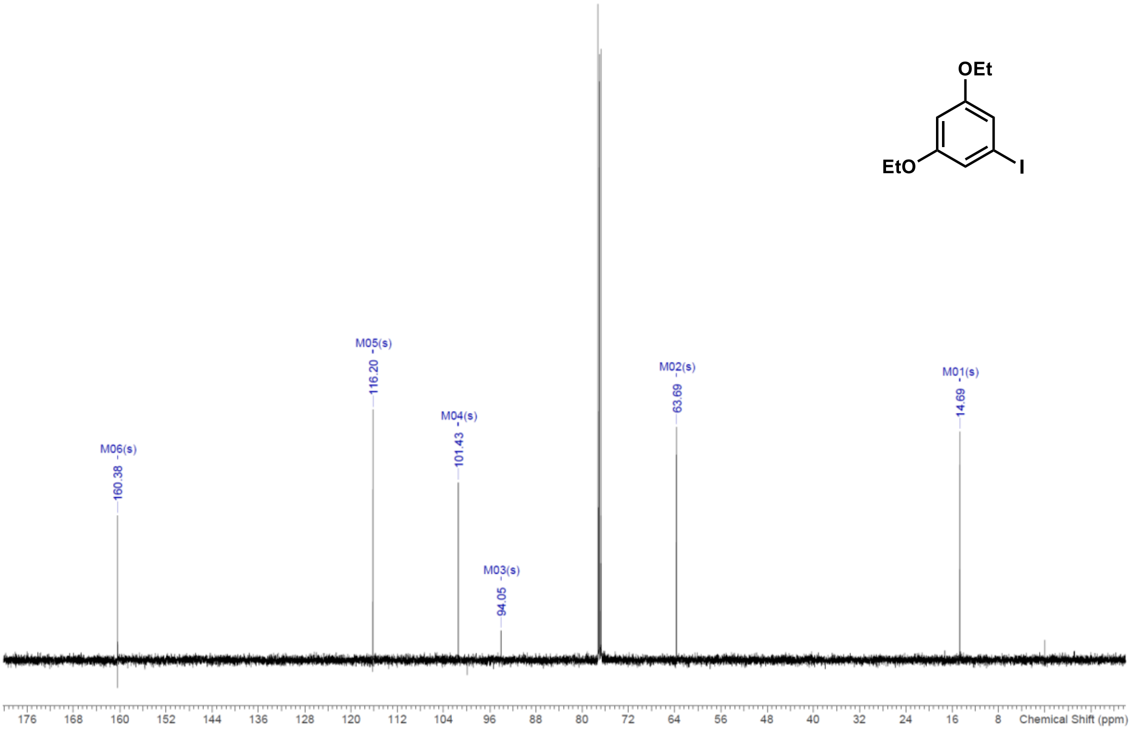

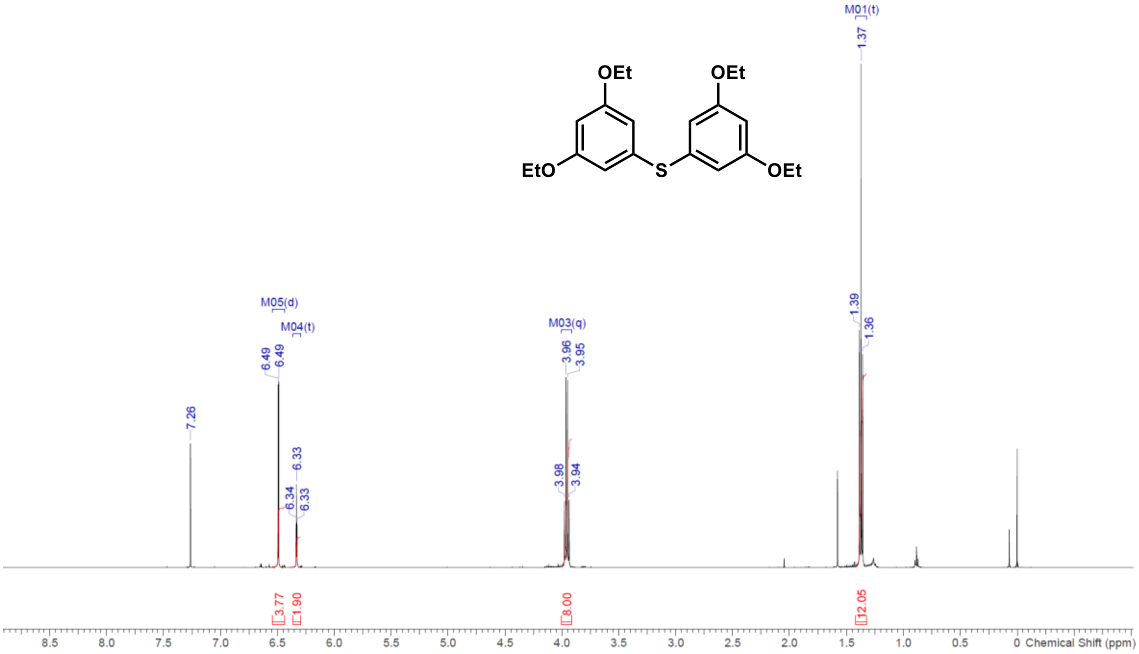

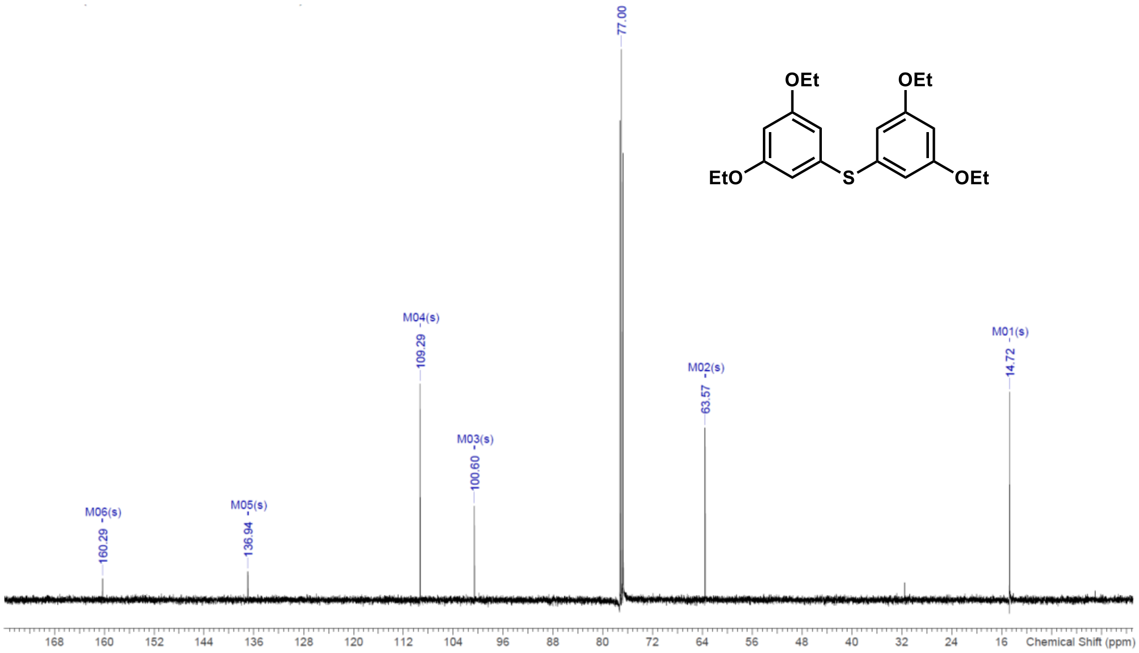

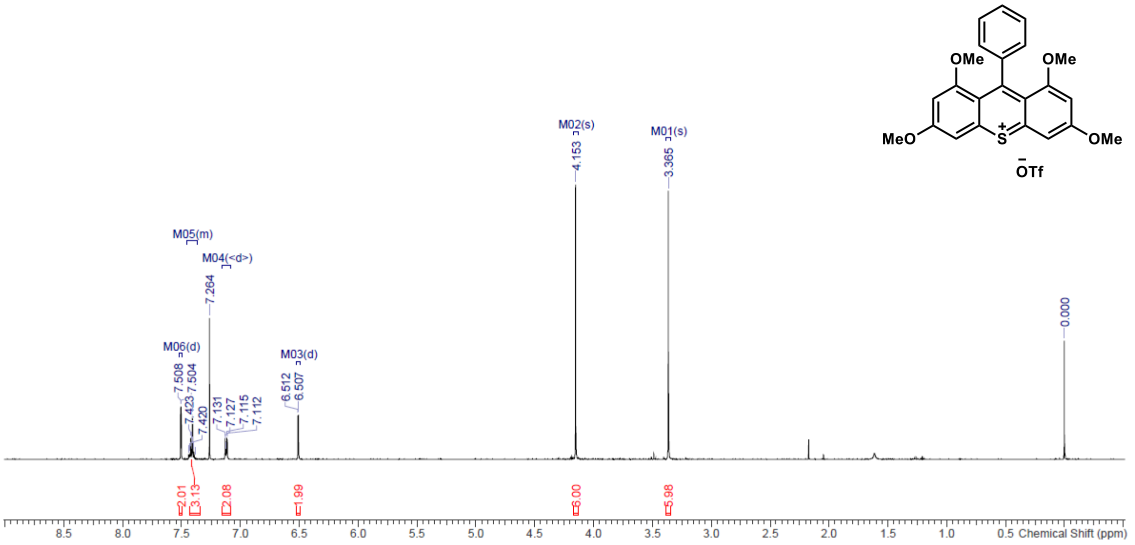

OMe
OMe
MeO
OMe
S+
OTf
M02(s)
4.153
M01(s)
3.365
M05(m)
M04(<d>)
7.264
M06(d)
7.508
7.504
7.423
7.420
7.131
7.127
7.115
7.112
M03(d)
6.512
6.507
0.000
2.01
3.13
2.08
1.99
6.00
5.98
Chemical Shift (ppm)

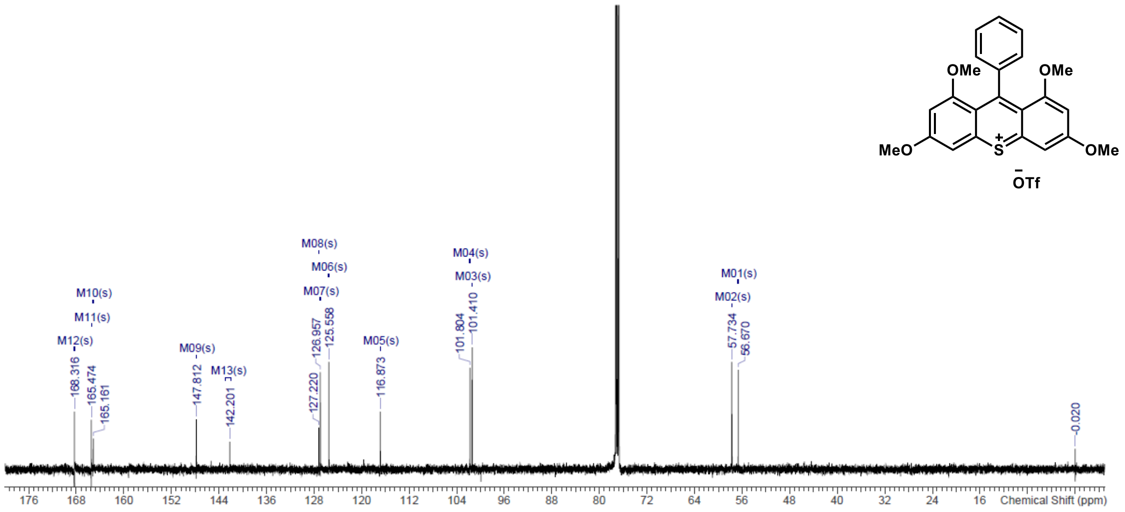

OMe
OMe
MeO
OMe
S+
OTf
M08(s)
M06(s)
M07(s)
126.957
125.558
127.220
M04(s)
M03(s)
101.804
101.410
M05(s)
116.873
M10(s)
M11(s)
M12(s)
168.316
165.474
165.161
M09(s)
147.812
M13(s)
142.201
M01(s)
M02(s)
57.734
56.670
-0.020
Chemical Shift (ppm)

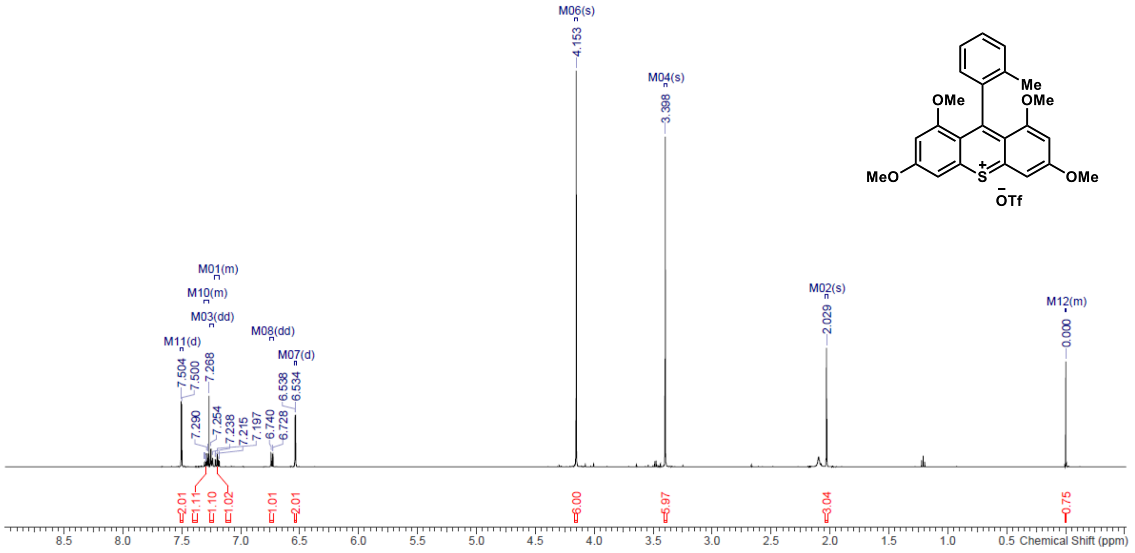

Me
OMe
OMe
MeO
OMe
S+
OTf
M06(s)
4.153
M04(s)
3.398
M02(s)
2.029
M12(m)
0.000
M01(m)
M10(m)
M03(dd)
M11(d)
M08(dd)
M07(d)
7.504
7.500
7.268
7.290
7.254
7.238
7.215
7.197
6.740
6.728
6.538
6.534
2.01
1.11
1.10
1.02
1.01
2.01
6.00
5.97
3.04
0.75
Chemical Shift (ppm)

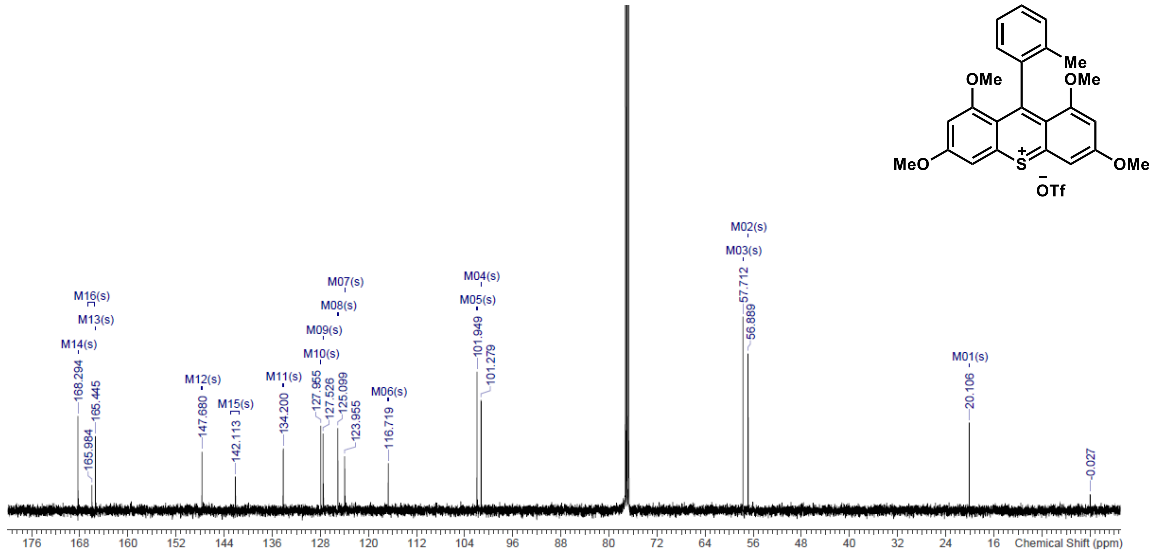
Me
OMe
OMe
MeO
OMe
S
OTf
168.294
165.984
165.445
147.680
142.113
134.200
127.955
127.526
125.099
123.955
116.719
101.949
101.279
57.712
56.889
20.106
0.027
Chemical Shift (ppm)

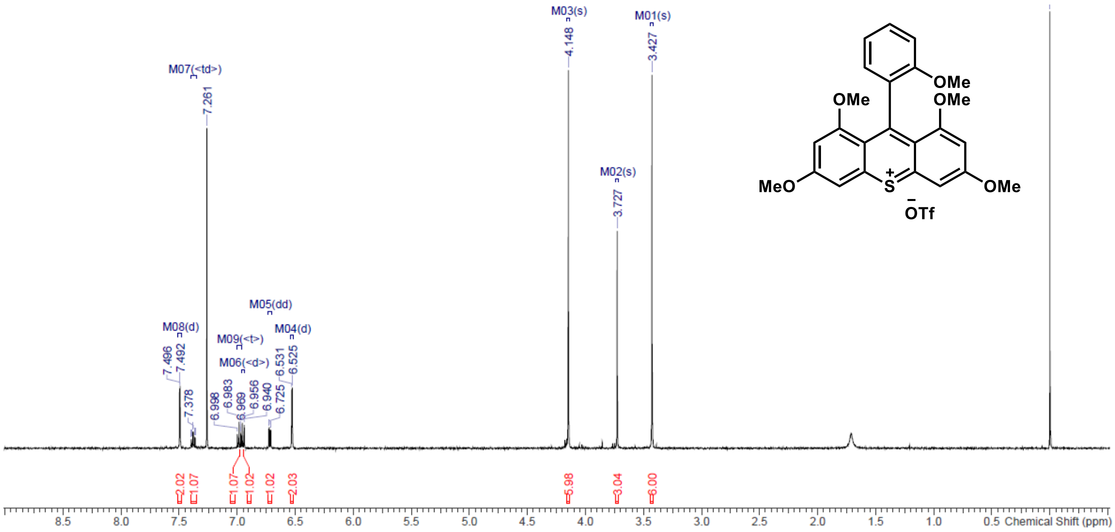
OMe
OMe
OMe
MeO
OMe
S
OTf
7.496
7.492
7.378
7.261
6.998
6.983
6.969
6.956
6.940
6.725
6.531
6.525
4.148
3.727
3.427
2.02
1.07
1.07
1.02
1.02
2.03
5.98
3.04
6.00
Chemical Shift (ppm)

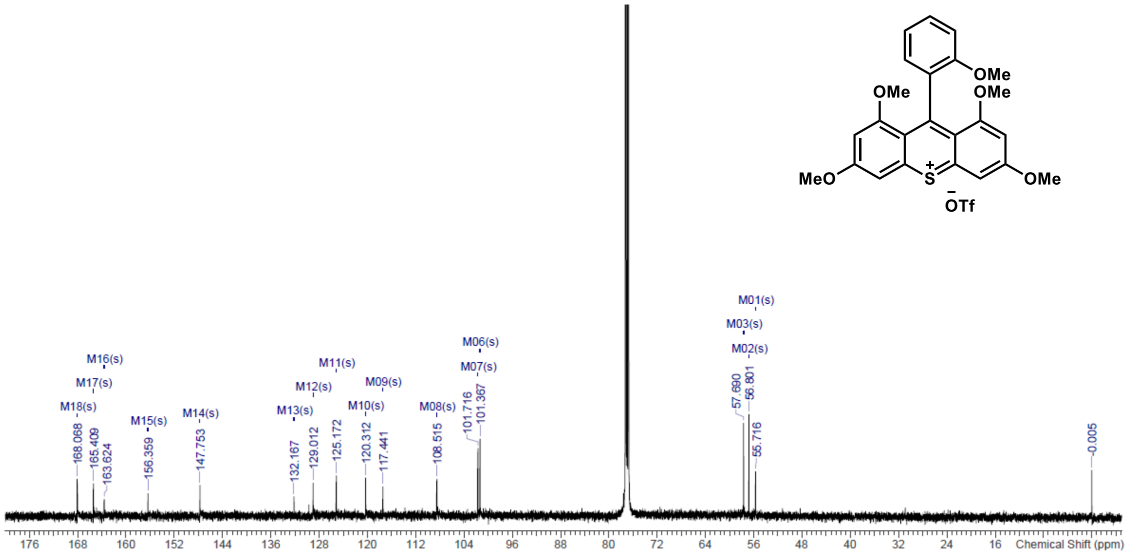
OMe
OMe
OMe
MeO
OMe
S
OTf
168.068
165.409
163.624
156.359
147.753
132.167
129.012
125.172
120.312
117.441
108.515
101.716
101.367
57.690
56.801
55.716
0.005
Chemical Shift (ppm)

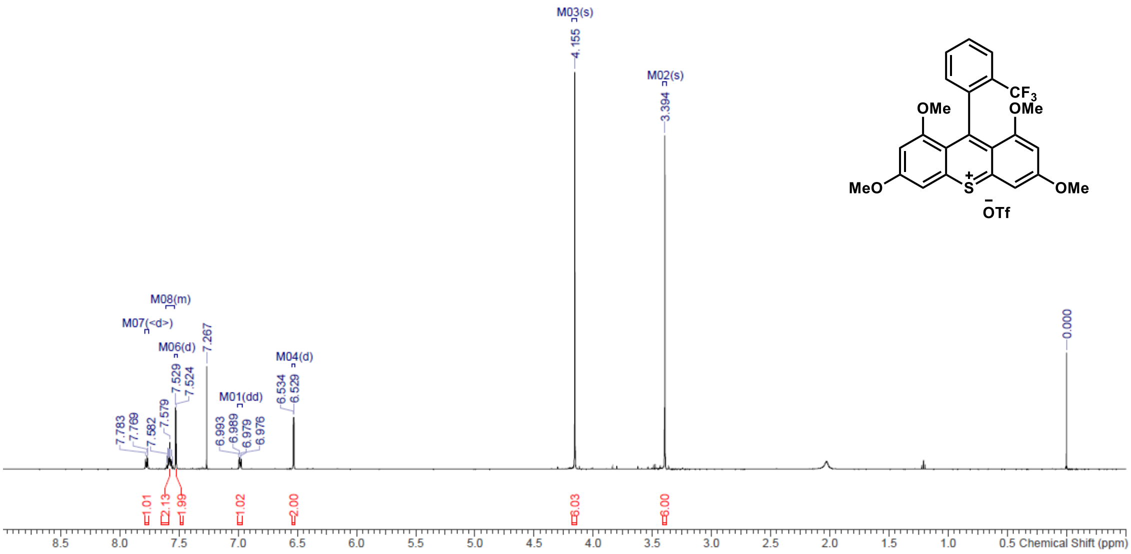
M03(s)
4.155
M02(s)
3.394
M08(m)
M07(<d>)
M06(d)
7.267
M04(d)
M01(dd)
7.783
7.769
7.582
7.579
7.529
7.524
6.993
6.989
6.979
6.976
6.534
6.529
0.000
1.01
2.13
1.99
1.02
2.00
6.03
6.00
Chemical Shift (ppm)
CF3
OMe
OMe
MeO
OMe
S
OTf

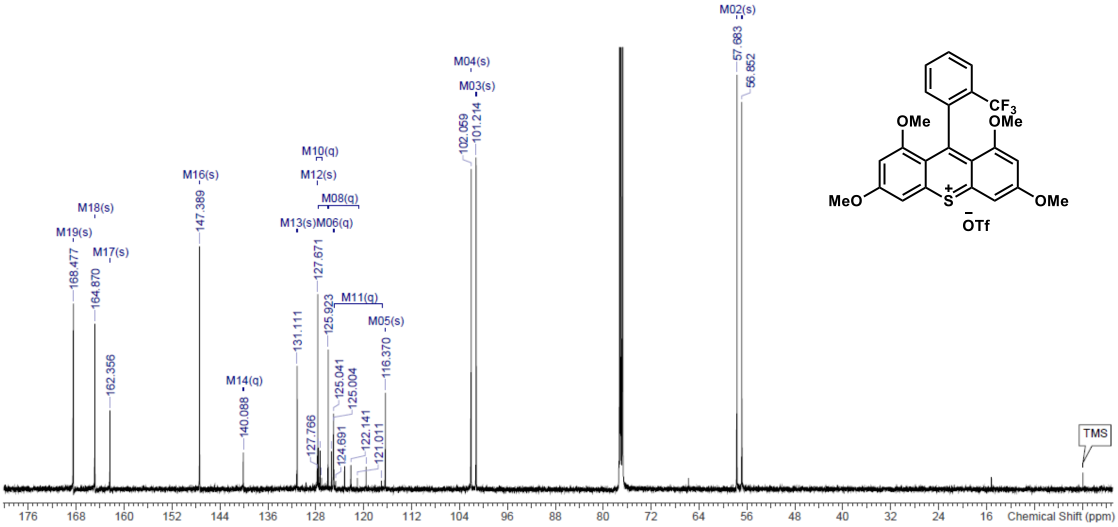
M02(s)
57.683
56.852
M04(s)
M03(s)
102.059
101.214
M10(q)
M12(s)
M08(q)
M13(s)
M06(q)
M16(s)
147.389
M18(s)
M19(s)
M17(s)
168.477
164.870
162.356
127.671
M11(q)
M05(s)
131.111
125.923
116.370
M14(q)
140.088
127.766
125.041
125.004
124.691
122.141
121.011
TMS
Chemical Shift (ppm)
CF3
OMe
OMe
MeO
OMe
S
OTf

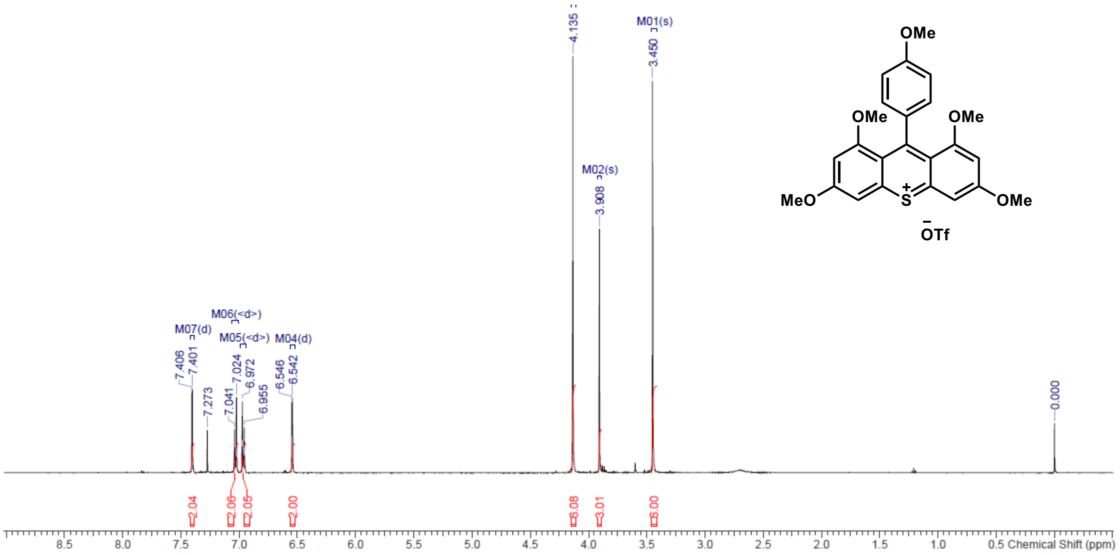
4.135
M01(s)
3.450
M02(s)
3.908
M06(<d>)
M07(d)
M05(<d>)
M04(d)
7.406
7.401
7.273
7.041
7.024
6.972
6.955
6.546
6.542
0.000
2.04
2.06
2.05
2.00
6.08
3.01
6.00
Chemical Shift (ppm)
OMe
OMe
OMe
MeO
OMe
S
OTf

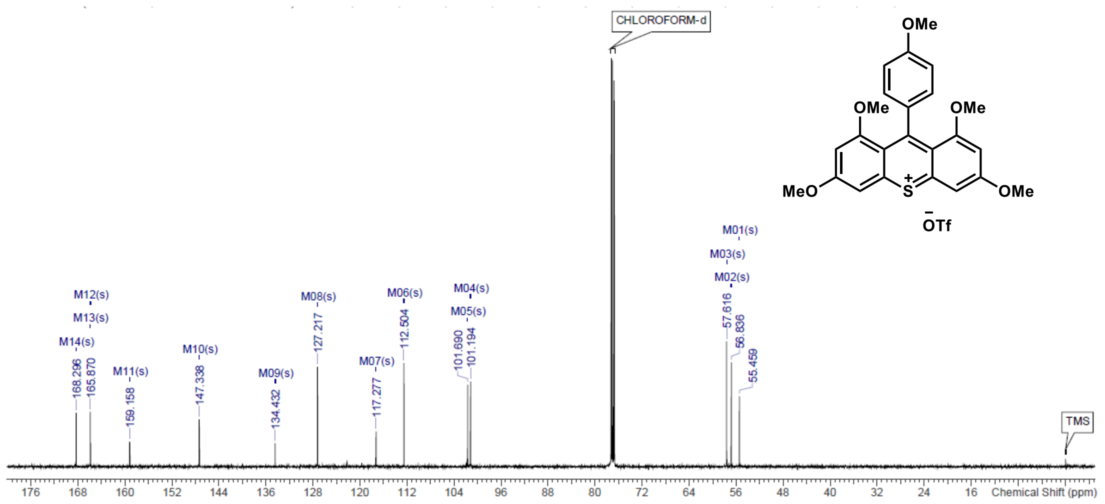
CHLOROFORM-d
OMe
OMe
OMe
MeO
OMe
S
OTf
M01(s)
M03(s)
M02(s)
57.616
56.836
55.459
M12(s)
M13(s)
M14(s)
168.296
165.870
M11(s)
159.158
M10(s)
147.338
M09(s)
134.432
M08(s)
127.217
M07(s)
117.277
M06(s)
112.504
M04(s)
M05(s)
101.690
101.194
TMS
Chemical Shift (ppm)

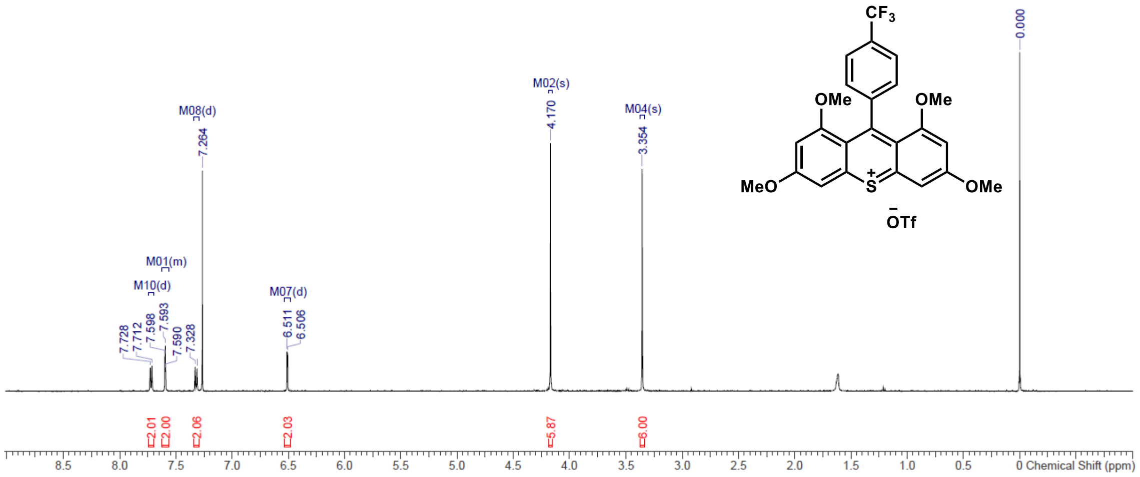
CF3
OMe
OMe
MeO
OMe
S
OTf
0.000
M02(s)
4.170
M04(s)
3.354
M08(d)
7.264
M01(m)
M10(d)
7.728
7.712
7.598
7.593
7.590
7.328
M07(d)
6.511
6.506
2.01
2.00
2.06
2.03
5.87
6.00
Chemical Shift (ppm)

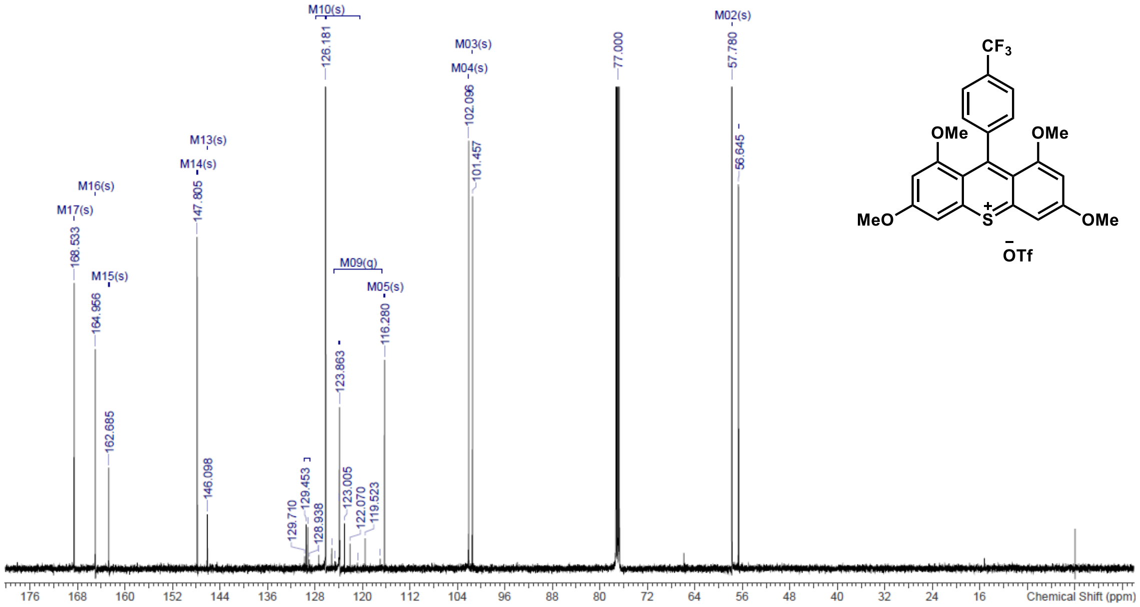
M10(s)
126.181
M03(s)
M04(s)
102.096
101.457
77.000
M02(s)
57.780
56.645
CF3
OMe
OMe
MeO
OMe
S
OTf
M13(s)
M14(s)
147.805
M16(s)
M17(s)
168.533
M15(s)
164.956
162.685
146.098
M09(q)
M05(s)
116.280
123.863
129.710
129.453
128.938
123.005
122.070
119.523
Chemical Shift (ppm)

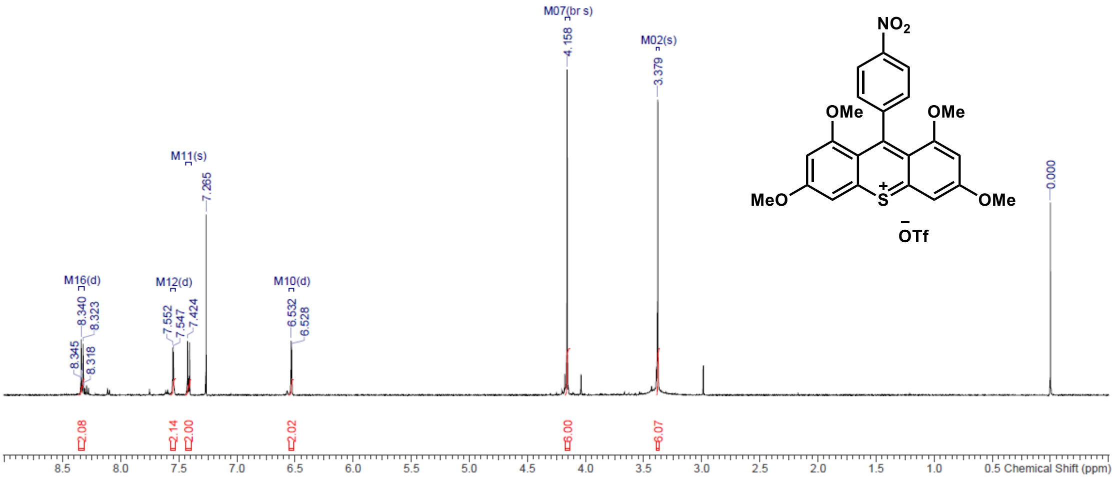
M07(br s)
4.158
M02(s)
3.379
M11(s)
7.265
M16(d)
8.345
8.340
8.323
8.318
M12(d)
7.552
7.547
7.424
M10(d)
6.532
6.528
0.000
2.08
2.14
2.00
2.02
6.00
6.07
Chemical Shift (ppm)
NO2
OMe
OMe
MeO
OMe
S
OTf

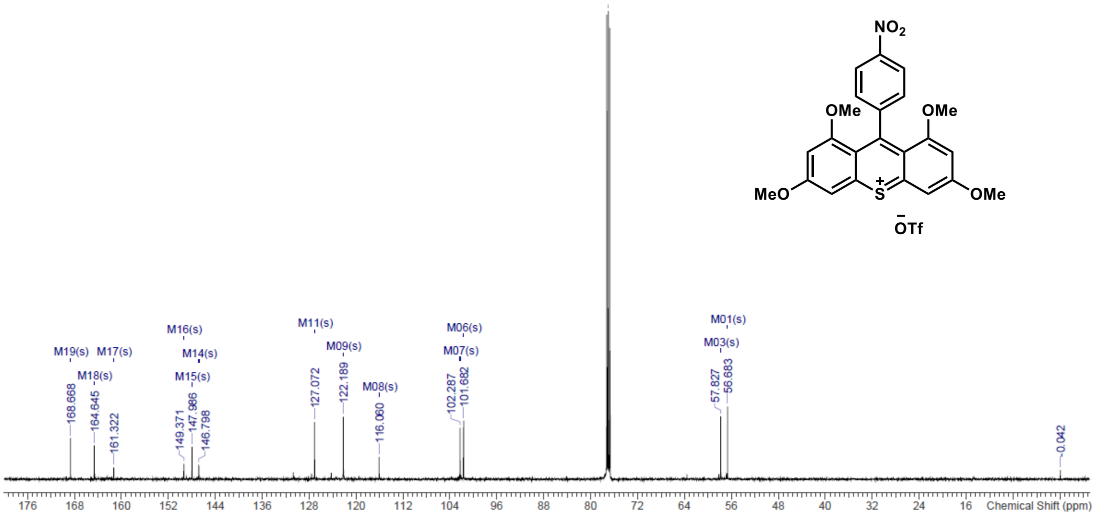
M19(s)
M17(s)
M18(s)
168.668
164.645
161.322
M16(s)
M14(s)
M15(s)
149.371
147.986
146.798
M11(s)
127.072
M09(s)
122.189
M08(s)
116.060
M06(s)
M07(s)
102.287
101.682
M01(s)
M03(s)
57.827
56.683
0.042
Chemical Shift (ppm)
NO2
OMe
OMe
MeO
OMe
S
OTf

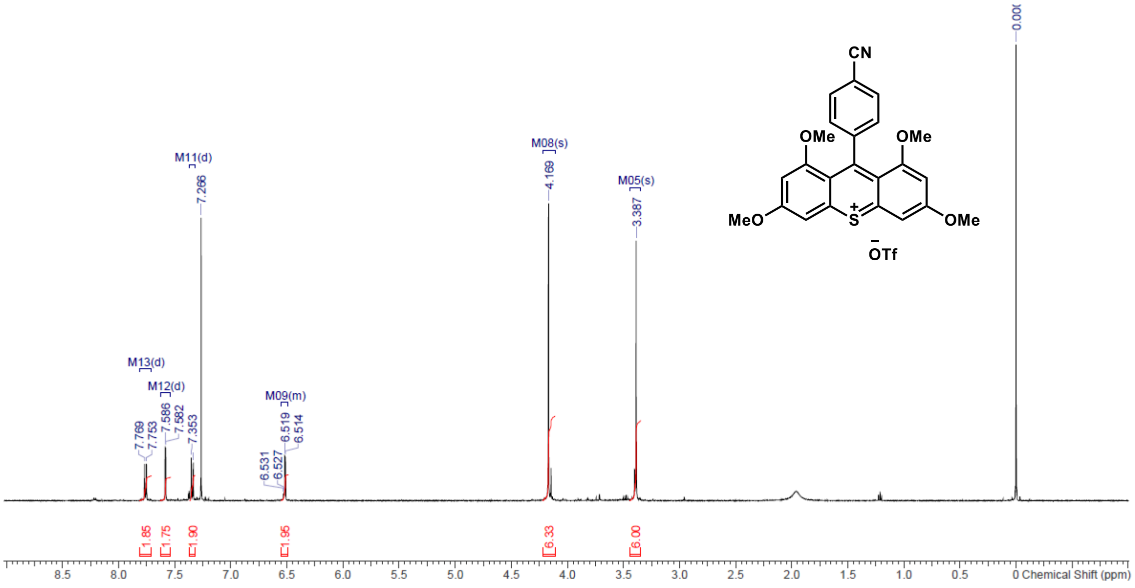
CN
OMe
OMe
MeO
OMe
OTf
M13(d)
M12(d)
M11(d)
M09(m)
M08(s)
M05(s)
7.769
7.753
7.586
7.582
7.353
7.266
6.531
6.527
6.519
6.514
4.169
3.387
0.000
1.85
1.75
1.90
1.95
6.33
6.00
Chemical Shift (ppm)

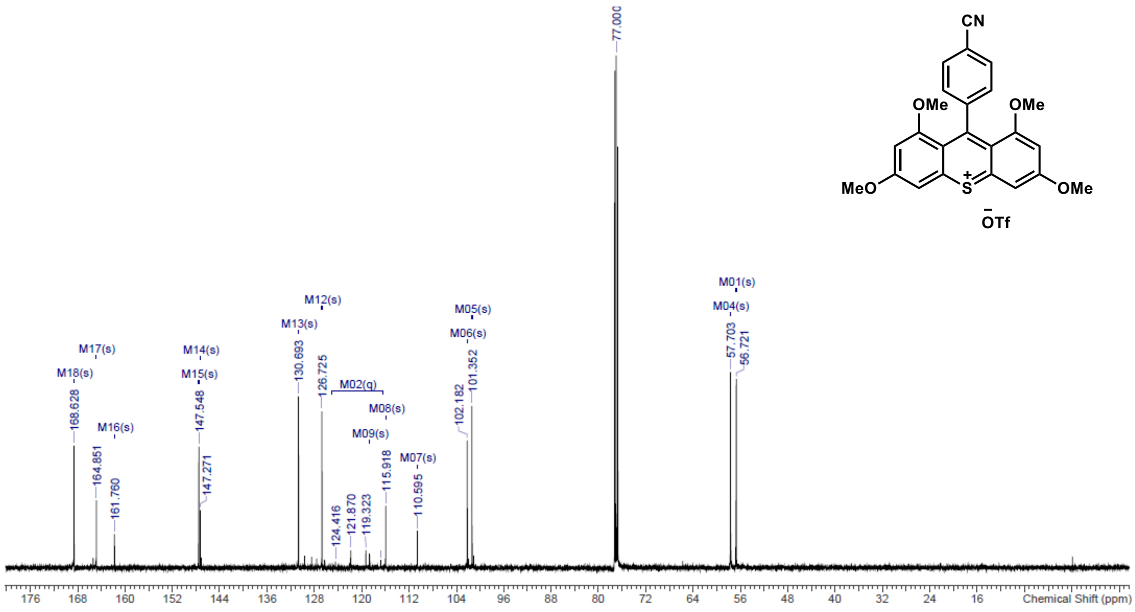
CN
OMe
OMe
MeO
OMe
OTf
M18(s)
M17(s)
M16(s)
M14(s)
M15(s)
M13(s)
M12(s)
M02(q)
M08(s)
M09(s)
M07(s)
M05(s)
M06(s)
M01(s)
M04(s)
168.628
164.851
161.760
147.548
147.271
130.693
126.725
124.416
121.870
119.323
115.918
110.595
102.182
101.352
77.000
57.703
56.721
Chemical Shift (ppm)

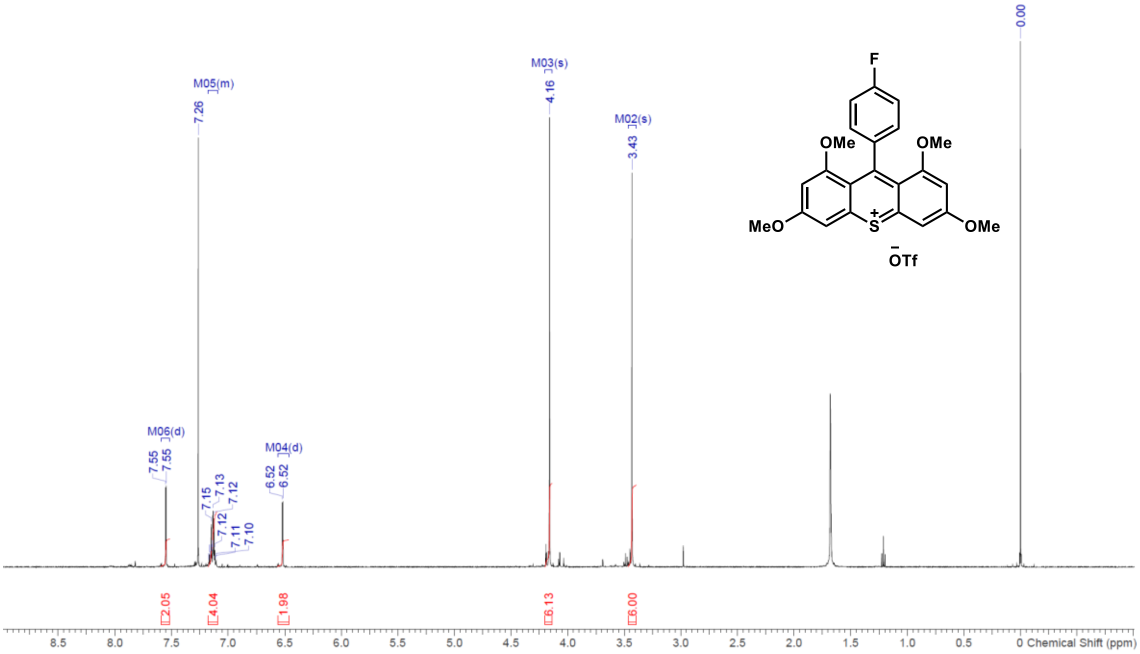
F
OMe
OMe
MeO
OMe
S
OTf
M05(m)
7.26
M03(s)
4.16
M02(s)
3.43
0.00
M06(d)
7.55
7.55
M04(d)
6.52
6.52
7.15
7.13
7.12
7.12
7.11
7.10
2.05
4.04
1.98
6.13
6.00
Chemical Shift (ppm)

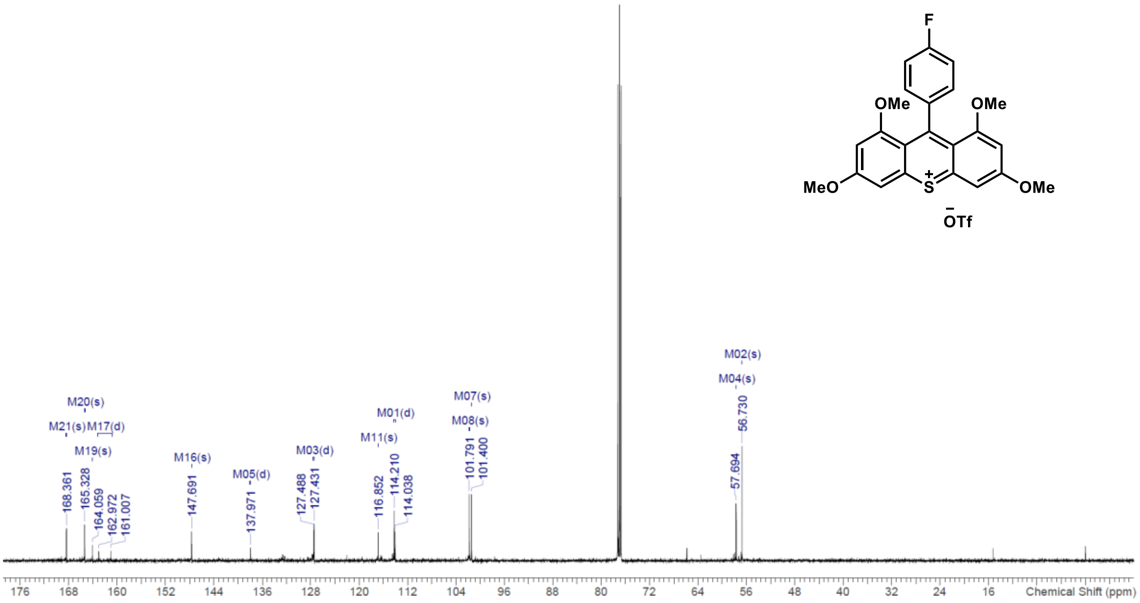
F
OMe
OMe
MeO
OMe
S
OTf
M20(s)
M21(s)
M17(d)
M19(s)
168.361
165.328
164.059
162.972
161.007
M16(s)
147.691
M05(d)
137.971
M03(d)
127.488
127.431
M01(d)
M11(s)
116.852
114.210
114.038
M07(s)
M08(s)
101.791
101.400
M02(s)
M04(s)
56.730
57.694
Chemical Shift (ppm)

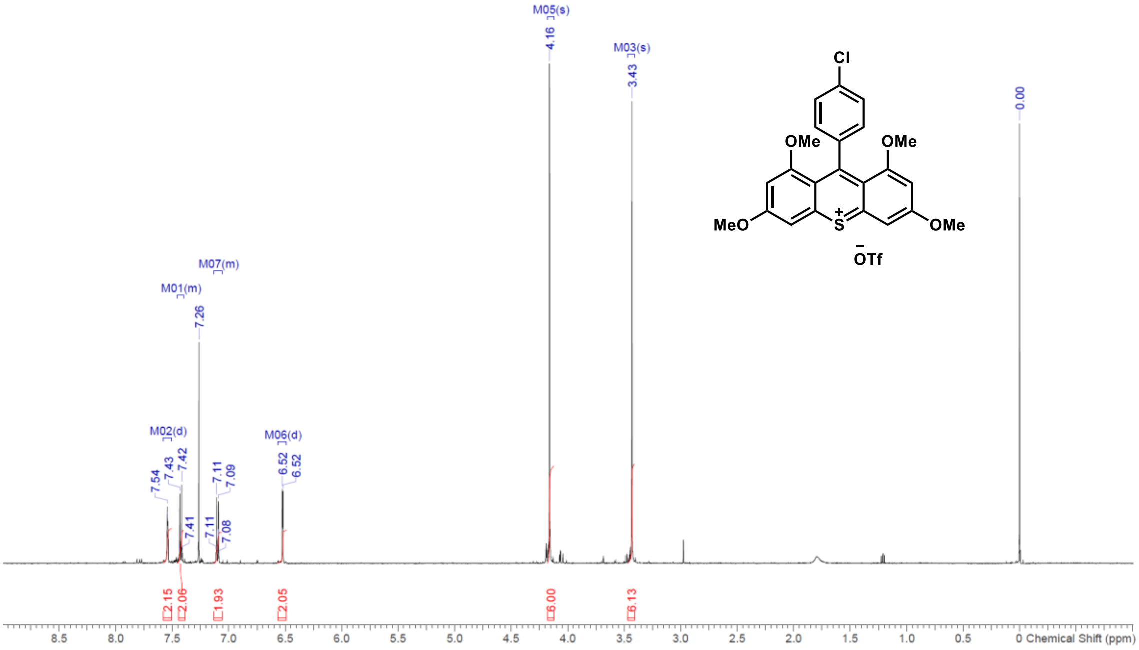
M05(s)
4.16
M03(s)
3.43
0.00
M07(m)
M01(m)
7.26
M02(d)
7.54
7.43
7.42
7.41
7.11
7.11
7.09
7.08
M06(d)
6.52
6.52
2.15
2.06
1.93
2.05
6.00
6.13
Cl
OMe
OMe
MeO
OMe
S
OTf
Chemical Shift (ppm)

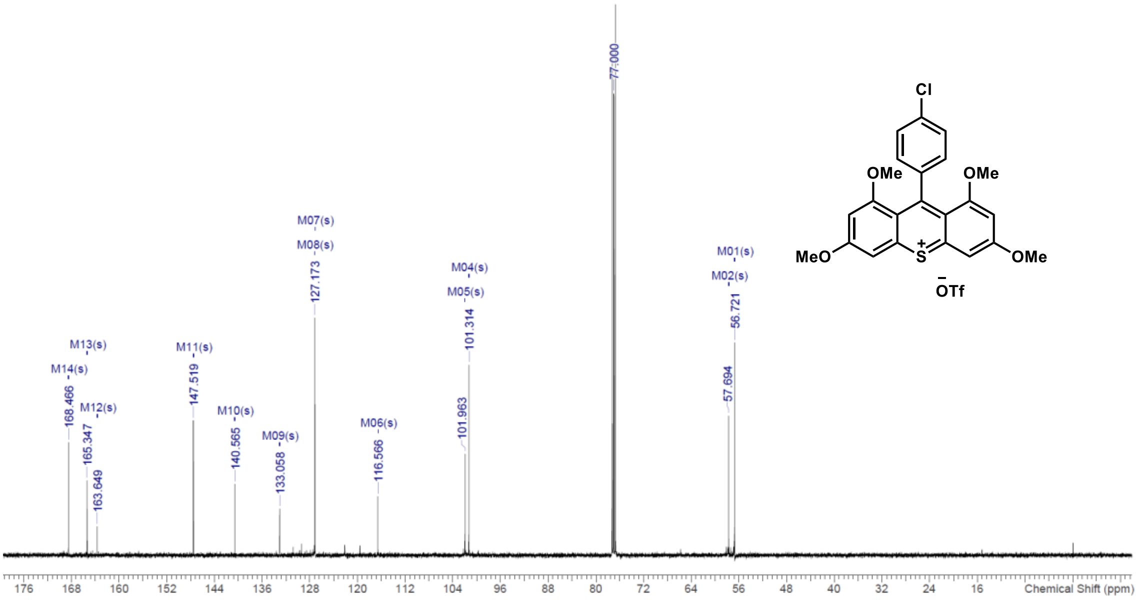
77.000
M07(s)
M08(s)
127.173
M04(s)
M05(s)
101.314
101.963
M01(s)
M02(s)
56.721
57.694
M13(s)
M14(s)
168.466
M12(s)
165.347
163.649
M11(s)
147.519
M10(s)
140.565
M09(s)
133.058
M06(s)
116.566
Cl
OMe
OMe
MeO
OMe
S
OTf
Chemical Shift (ppm)

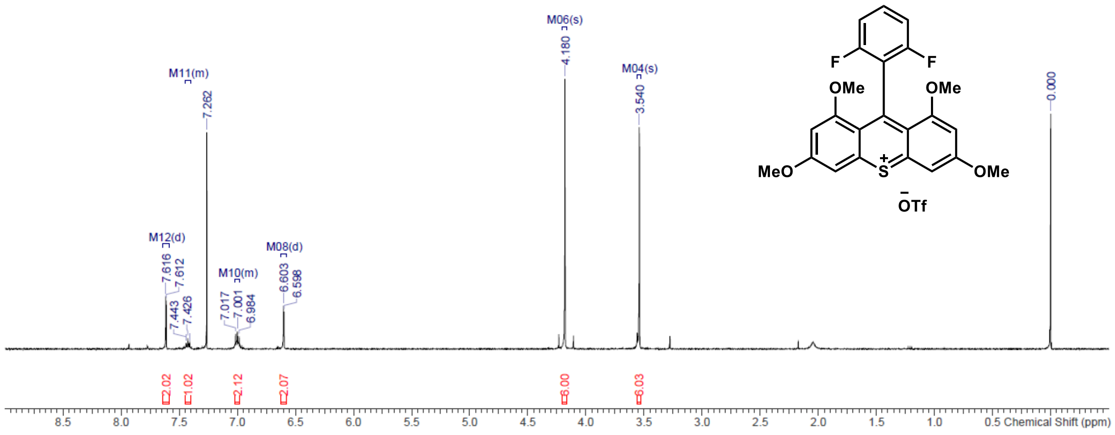
M06(s)
4.180
M04(s)
3.540
M11(m)
7.262
M12(d)
7.616
7.612
7.443
7.426
M10(m)
7.017
7.001
6.984
M08(d)
6.603
6.598
0.000
2.02
1.02
2.12
2.07
6.00
6.03
Chemical Shift (ppm)
F
F
OMe
OMe
MeO
OMe
S
OTf

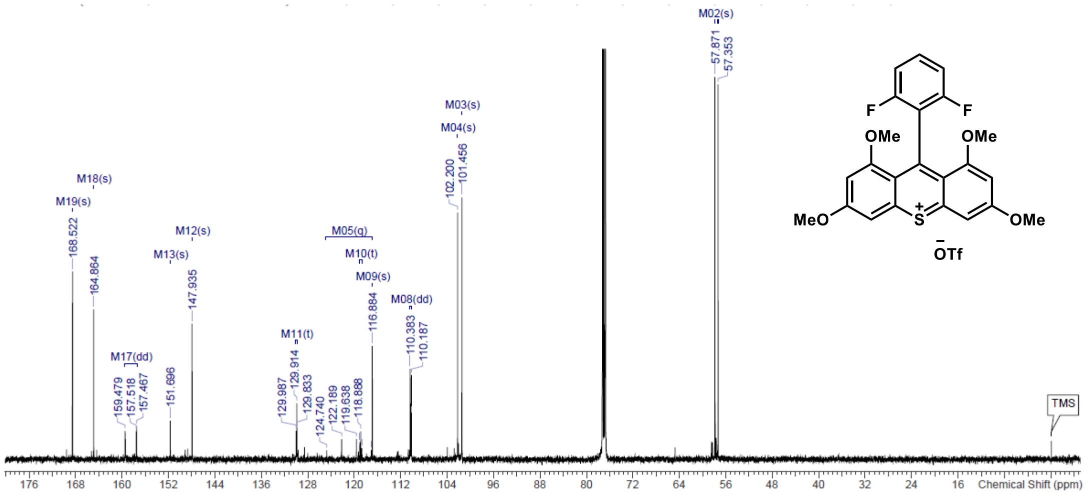
M02(s)
57.871
57.353
M03(s)
M04(s)
102.200
101.456
M18(s)
M19(s)
168.522
164.864
M12(s)
M13(s)
147.935
151.696
M17(dd)
159.479
157.518
157.467
M05(q)
M10(t)
M09(s)
116.884
M08(dd)
110.383
110.187
M11(t)
129.987
129.914
129.833
124.740
122.189
119.638
118.888
TMS
Chemical Shift (ppm)
F
F
OMe
OMe
MeO
OMe
S
OTf

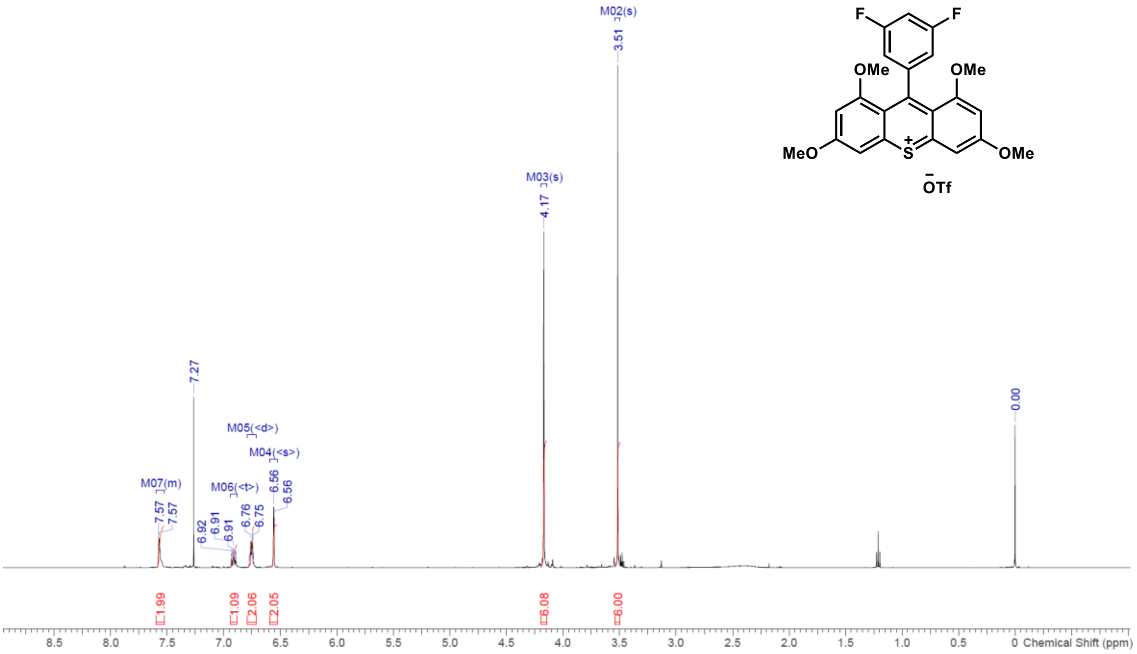
M02(s)
3.51
M03(s)
4.17
7.27
0.00
M05(<d>)
M04(<s>)
M07(m)
M06(<t>)
7.57
7.57
6.92
6.91
6.91
6.76
6.75
6.56
6.56
1.99
1.09
2.06
2.05
6.08
6.00
Chemical Shift (ppm)
F
F
OMe
OMe
MeO
OMe
S
OTf

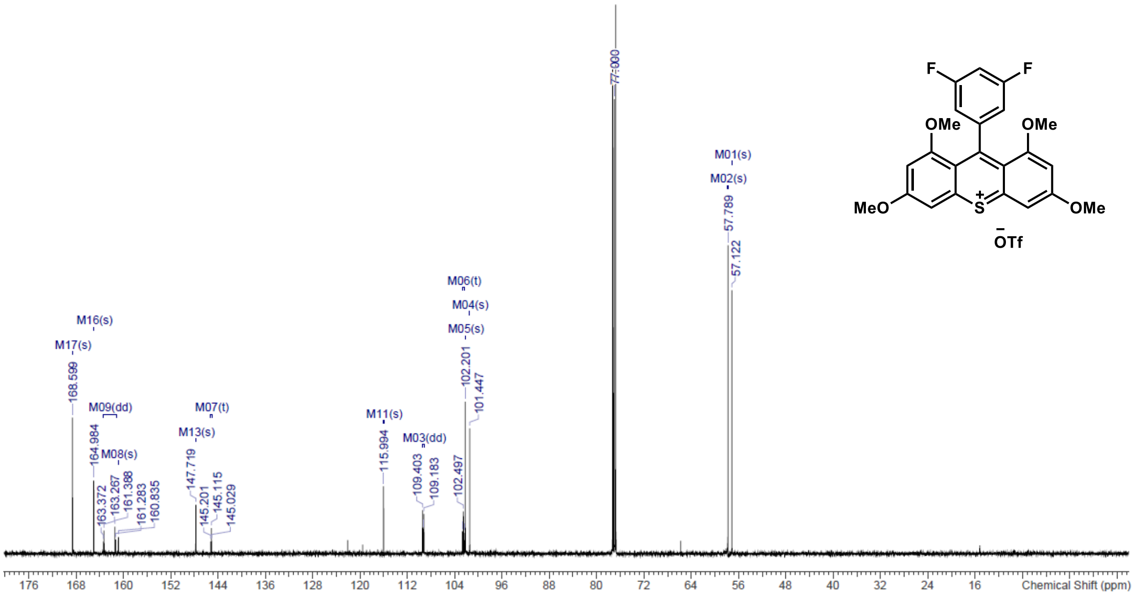
77.000
M01(s)
M02(s)
57.789
57.122
M06(t)
M04(s)
M05(s)
102.201
101.447
M16(s)
M17(s)
168.599
M09(dd)
M07(t)
M13(s)
M11(s)
M03(dd)
164.984
M08(s)
163.372
163.267
161.388
161.283
160.835
147.719
145.201
145.115
145.029
115.994
109.403
109.183
102.497
Chemical Shift (ppm)
F
F
OMe
OMe
MeO
OMe
S
OTf

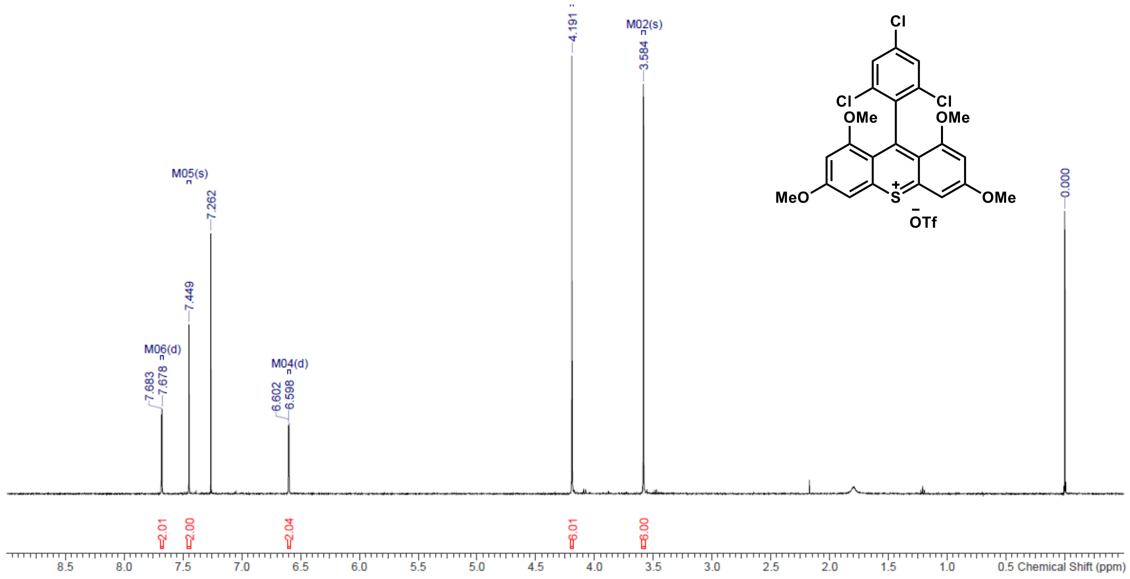
M05(s)
M06(d)
M04(d)
M02(s)
7.683
7.678
7.449
7.262
6.602
6.598
4.191
3.584
0.000
2.01
2.00
2.04
6.01
6.00
Cl
OMe
MeO
S
OTf
Chemical Shift (ppm)

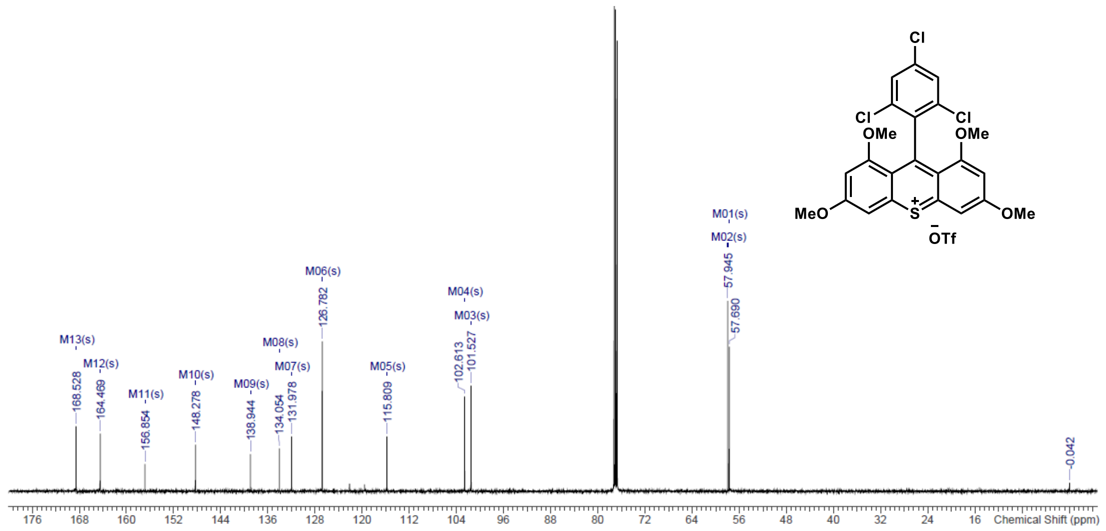
M13(s)
M12(s)
M11(s)
M10(s)
M09(s)
M08(s)
M07(s)
M06(s)
M05(s)
M04(s)
M03(s)
M01(s)
M02(s)
168.528
164.469
156.854
148.278
138.944
134.054
131.978
126.782
115.809
102.613
101.527
57.945
57.690
0.042
Cl
OMe
MeO
S
OTf
Chemical Shift (ppm)

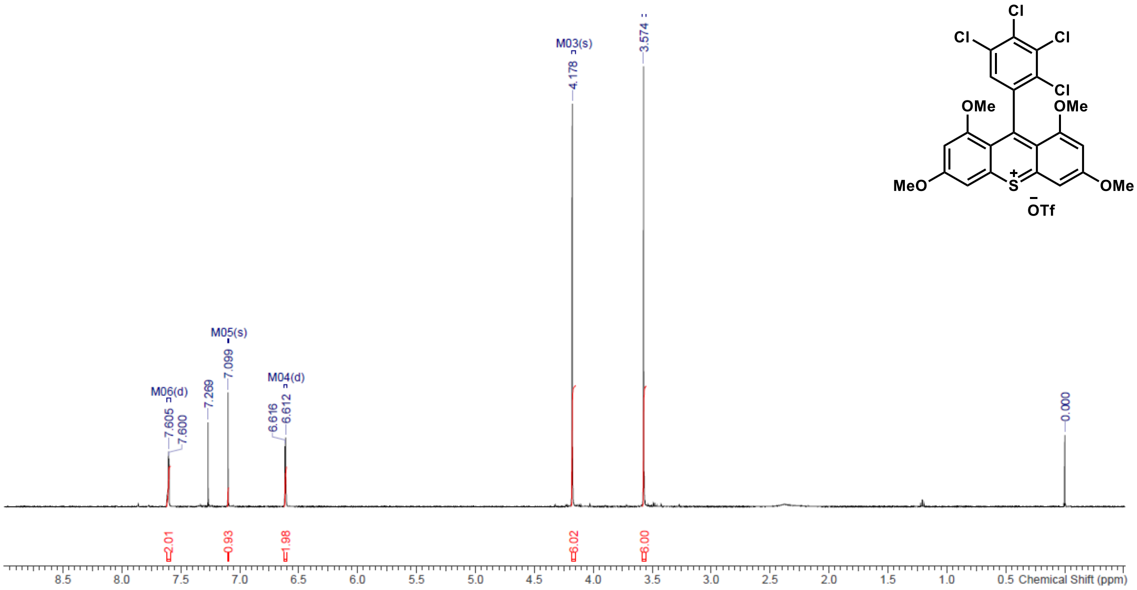
Cl
Cl
Cl
Cl
OMe
OMe
MeO
OMe
S
OTf
M03(s)
4.178
3.574
M05(s)
7.099
M06(d)
7.605
7.600
7.269
M04(d)
6.616
6.612
0.000
2.01
0.93
1.98
6.02
6.00
8.5
8.0
7.5
7.0
6.5
6.0
5.5
5.0
4.5
4.0
3.5
3.0
2.5
2.0
1.5
1.0
0.5 Chemical Shift (ppm)

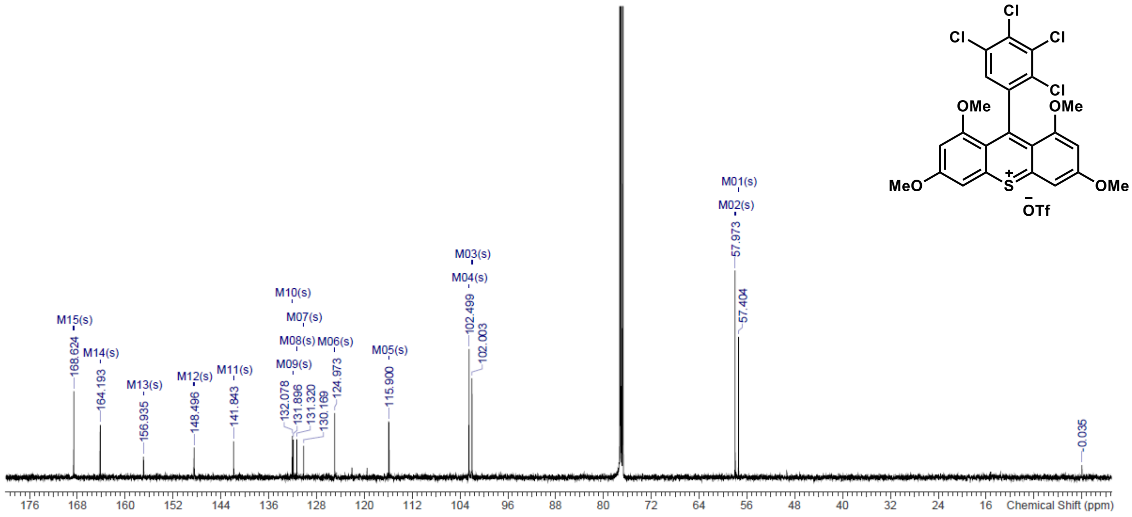
Cl
Cl
Cl
Cl
OMe
OMe
MeO
OMe
S
OTf
M01(s)
M02(s)
57.973
57.404
M03(s)
M04(s)
102.499
102.003
M10(s)
M07(s)
M08(s)
M06(s)
M09(s)
M05(s)
132.078
131.896
131.320
130.169
124.973
115.900
M15(s)
168.624
M14(s)
164.193
M13(s)
156.935
M12(s)
148.496
M11(s)
141.843
0.035
176
168
160
152
144
136
128
120
112
104
96
88
80
72
64
56
48
40
32
24
16
Chemical Shift (ppm)

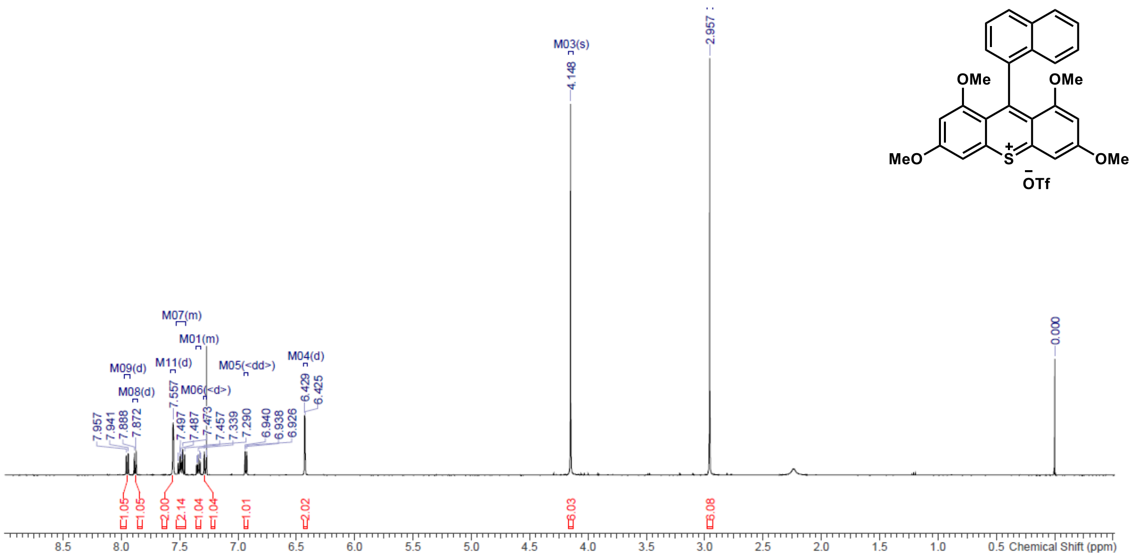

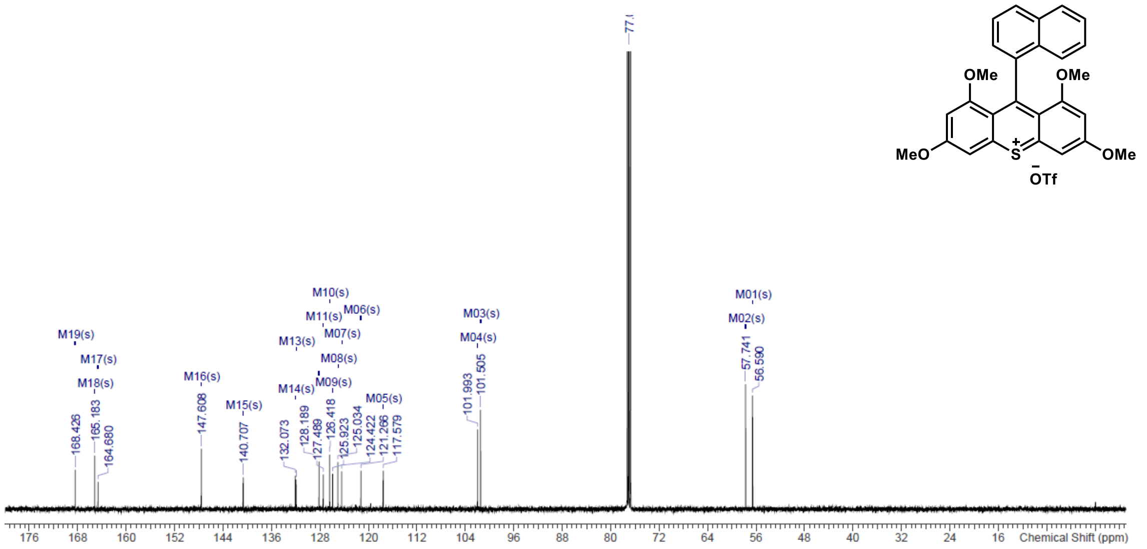

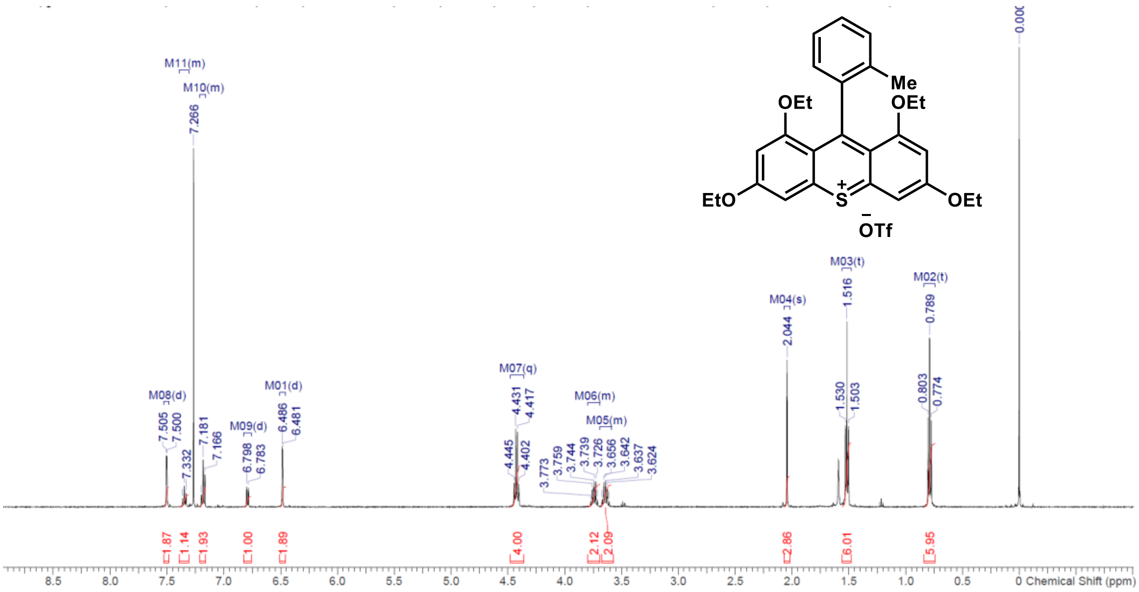

Me
OEt
OEt
EtO
OEt
OTf
0 Chemical Shift (ppm)

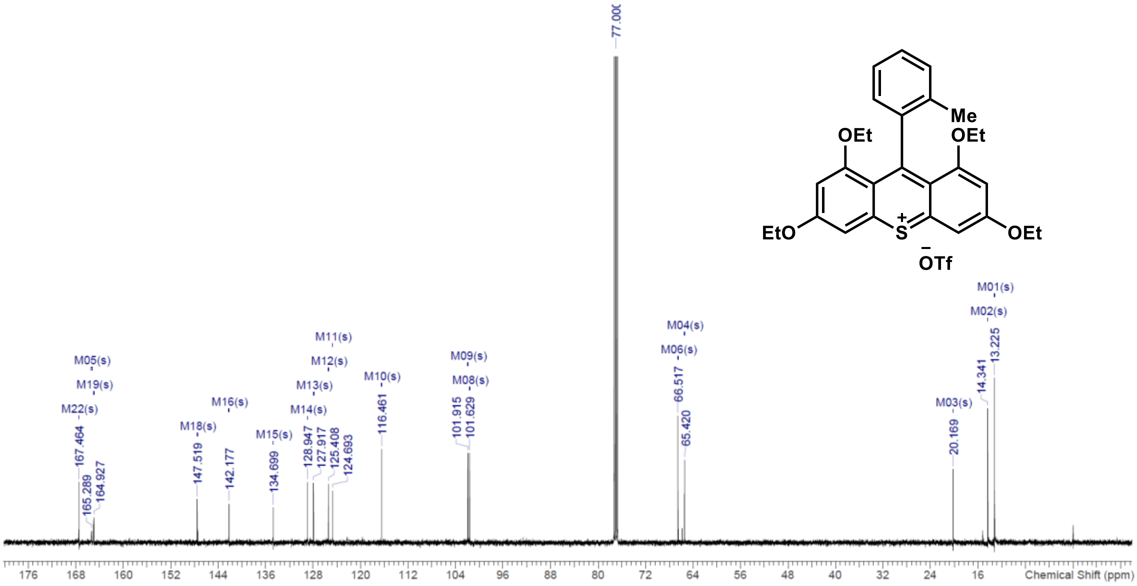

Me
OEt
OEt
EtO
OEt
OTf
Chemical Shift (ppm)

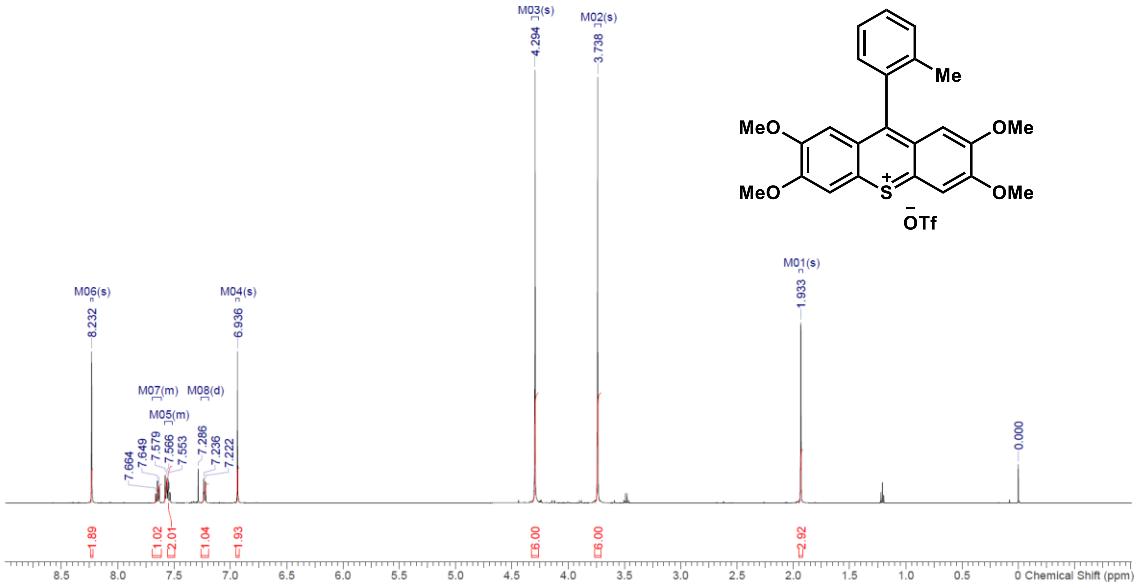
M03(s)
4.294
M02(s)
3.738
Me
MeO
OMe
MeO
OMe
S
OTf
M01(s)
1.933
M06(s)
8.232
M04(s)
6.936
M07(m)
M08(d)
M05(m)
7.664
7.649
7.579
7.566
7.553
7.286
7.236
7.222
0.000
1.89
1.02
2.01
1.04
1.93
6.00
6.00
2.92
Chemical Shift (ppm)

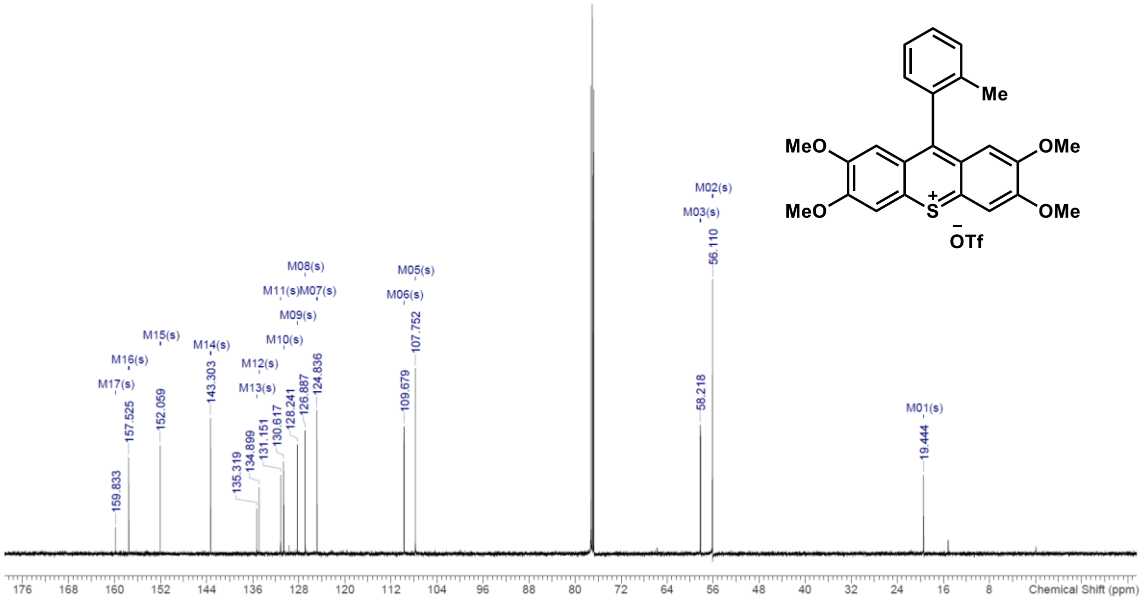
Me
MeO
OMe
MeO
OMe
S
OTf
M02(s)
M03(s)
56.110
58.218
M08(s)
M11(s)M07(s)
M09(s)
M10(s)
M05(s)
M06(s)
107.752
109.679
M15(s)
M14(s)
M16(s)
M17(s)
M12(s)
M13(s)
143.303
152.059
157.525
159.833
135.319
134.899
131.151
130.617
128.241
126.887
124.836
M01(s)
19.444
Chemical Shift (ppm)

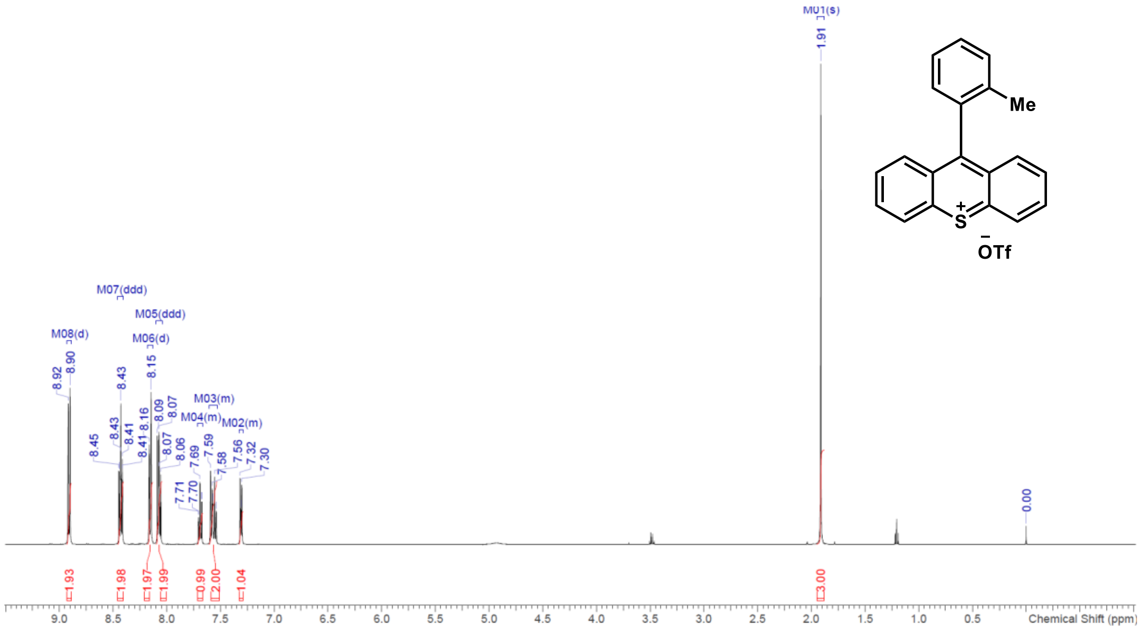

M01(s)
1.91
Me
S
OTf
M07(ddd)
M05(ddd)
M08(d)
M06(d)
8.92
8.90
8.43
8.15
8.45
8.43
8.41
8.41
8.16
8.09
8.07
8.07
8.06
M03(m)
M04(m)
M02(m)
7.71
7.70
7.69
7.59
7.58
7.56
7.32
7.30
0.00
1.93
1.98
1.97
1.99
0.99
2.00
1.04
3.00
Chemical Shift (ppm)

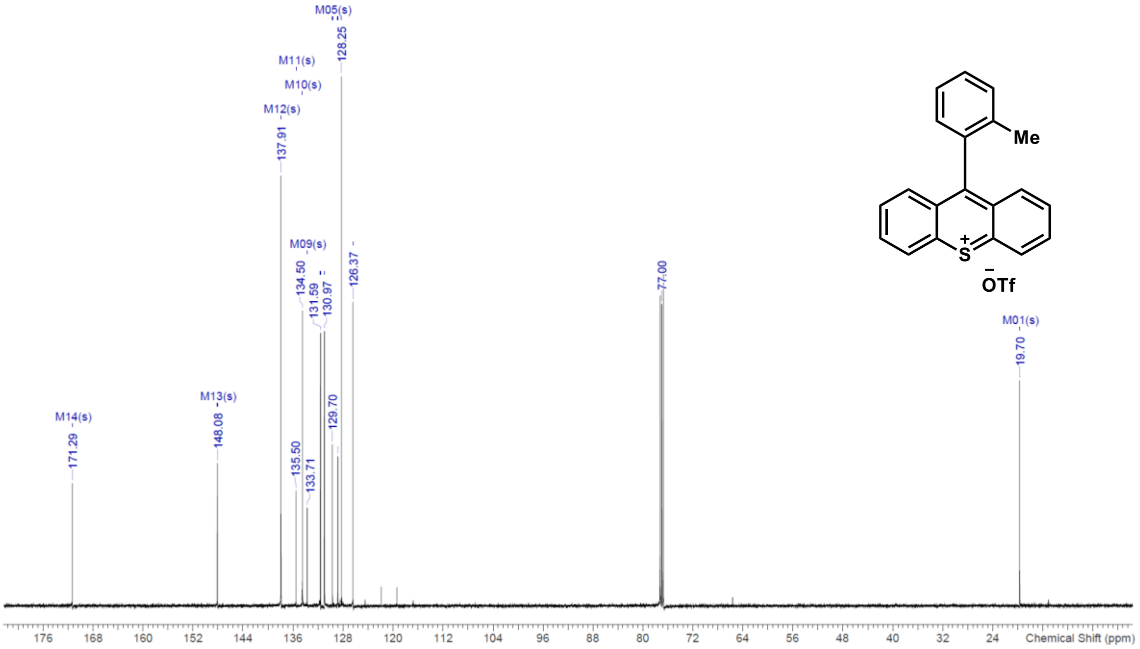

M05(s)
128.25
M11(s)
M10(s)
M12(s)
137.91
Me
S
OTf
M09(s)
134.50
131.59
130.97
126.37
77.00
M01(s)
19.70
M13(s)
M14(s)
148.08
171.29
135.50
133.71
129.70
Chemical Shift (ppm)
